# Exploring voltage-gated sodium channel conformations and protein-protein interactions using AlphaFold2

**DOI:** 10.1101/2024.10.15.618559

**Published:** 2024-10-18

**Authors:** Diego Lopez-Mateos, Kush Narang, Vladimir Yarov-Yarovoy

**Affiliations:** 1 Department of Physiology and Membrane Biology, University of California School of Medicine, Davis, CA 95616; 2 Biophysics Graduate Group, University of California School of Medicine, Davis, CA 95616; 3 Department of Anesthesiology and Pain Medicine, University of California School of Medicine, Davis, CA 95616

## Abstract

Voltage-gated sodium (Na_V_) channels are vital regulators of electrical activity in excitable cells, playing critical roles in generating and propagating action potentials. Given their importance in physiology, Na_V_ channels are key therapeutic targets for treating numerous conditions, yet developing subtype-selective drugs remains challenging due to the high sequence and structural conservation among Na_V_ family members. Recent advances in cryo-electron microscopy have resolved nearly all human Na_V_ channels, providing valuable insights into their structure and function. However, limitations persist in fully capturing the complex conformational states that underlie Na_V_ channel gating and modulation. This study explores the capability of AlphaFold2 to sample multiple Na_V_ channel conformations and assess AlphaFold Multimer’s accuracy in modeling interactions between the Na_V_ α-subunit and its protein partners, including auxiliary β-subunits and calmodulin. We enhance conformational sampling to explore Na_V_ channel conformations using a subsampled multiple sequence alignment approach and varying the number of recycles. Our results demonstrate that AlphaFold2 models multiple Na_V_ channel conformations, including those from experimental structures, new states not yet experimentally identified, and potential intermediate states. Furthermore, AlphaFold Multimer models Na_V_ complexes with auxiliary β-subunits and calmodulin with high accuracy, and the presence of protein partners significantly alters the conformational landscape of the Na_V_ α-subunit. These findings highlight the potential of deep learning-based methods to expand our understanding of Na_V_ channel structure, gating, and modulation, with significant implications for future drug discovery efforts.

## Introduction

Voltage-gated sodium (Na_V_) channels are essential regulators of electrical activity in excitable cells (Catterall, 2023). They open in response to membrane depolarization, allowing sodium ions to enter the cell and initiate transduction of sensory stimuli. This process is crucial for generating and propagating action potentials in neurons and muscle cells. Given their vital role in physiology, Na_V_ channels are targeted by numerous drugs for the treatment of various conditions such as epilepsy, pain or cardiac arrythmia (Bagal et al., 2013; Noreng et al., 2021), and mutations in these channels have been linked to a wide range of diseases (Bennett and Woods, 2014; Meisler et al., 2021; Pan et al., 2021).

The Na_V_ family in mammals contains nine subtypes, Na_V_1.1 through Na_V_1.9, with each exhibiting distinct tissue and cellular expression patterns that contribute to their specific physiological function (Catterall et al., 2020a). For instance, Na_V_1.2 is predominantly found in brain, Na_V_1.4 in skeletal muscle, Na_V_1.5 in cardiac myocytes, and Na_V_1.7 in nociceptive sensory neurons. Targeting and modulating specific Na_V_ subtypes has empowered therapeutic interventions for numerous pathologies, including cardiac arrhythmias (Remme and Wilde, 2014) and pain conditions (Alsaloum et al., 2020; Nguyen and Yarov-Yarovoy, 2022). However, achieving precise subtype selectivity—modulating a particular Na_V_ channel without affecting others—remains a significant challenge due to the high degree of overall sequence and structural conservation among the family members. As a result, many Na_V_-targeting drugs lack subtype selectivity, limiting their therapeutic applicability due to off-target side effects (Bagal et al., 2013). While notable advances have been made in developing subtype-selective modulators, such as Vertex’s VX-548, which inhibits human Na_V_1.8 with more than 30,000-fold selectivity over the other human Na_V_ subtypes (Jones et al., 2023), achieving high subtype selectivity for individual Na_V_ channels remains a critical challenge. Furthermore, due to the complex conformational states of Na_V_ channels, developing state-selective drugs—preferentially targeting channels in specific functional states—is equally important for precisely regulating their electrical activity.

Recent advances in cryo-electron microscopy (CryoEM) have enabled the resolution of almost all human Na_V_ channels, except for Na_V_1.9 (Pan et al., 2021, 2019; Li et al., 2022; Pan et al., 2018; Li et al., 2021a; Fan et al., 2023; Huang et al., 2022a; b). These breakthroughs have provided critical insights into the structure, function, and mechanisms of Na_V_ channels, building a robust foundation for future drug discovery efforts targeting this important membrane protein family. All mammalian Na_V_ channels share a conserved architecture, with a core α-subunit and auxiliary β-subunits (Catterall, 2023). The Na_V_ α-subunit consists of four homologous domains, each comprising six transmembrane segments (S1-S6). The first four segments (S1-S4) form the voltage-sensing domains (VSDs), while S5 and S6 form the pore domain (PD). The VSDs are positioned outwards from the pore, while the PD forms the central ion-conducting pore. The S4 segments, which carry the gating charges, respond to changes in membrane potential by conformational changes between deactivated and activated states, driving the channel’s opening, inactivation, and closing. Auxiliary Na_V_ β-subunits (β1-β4) are transmembrane proteins with extracellular immunoglobulin domains that interact with the Na_V_ α-subunit to modulate various channel properties (Namadurai et al., 2015). These include surface localization, kinetic behavior, and clustering, making them crucial regulators of cellular excitability. Additionally, Na_V_ channels are modulated by calcium through calmodulin (CaM) binding (Wu and Hong, 2021). Moreover, several peptide toxins from animal venoms, such as Protoxin-II and α-scorpion toxins, modulate Na_V_ channel activity (Bosmans and Tytgat, 2007; Sokolov et al., 2008). Recent cryoEM and X-ray structures of Na_V_ channels in complex with multiple modulators have further illuminated their modulatory mechanisms, offering valuable structural insights for drug development campaigns targeting Na_V_ channels (Shen et al., 2018; Clairfeuille et al., 2019; Shen et al., 2019; Jiang et al., 2021b; Li et al., 2024).

A growing body of structural and functional data has significantly advanced our understanding of the Na_V_ conformational cycle and regulation (Catterall et al., 2020b). In the resting state, the channels are closed, and the VSDs are deactivated, with the S4 segments in a “down” position. Upon membrane depolarization, the change in the magnitude and direction of the electric field exerts force on the positively charged gating charges within the VSDs, causing the S4 segments to shift “up” and transition the VSDs to an activated state. This mechanical work is transmitted to the activation gate (AG), a constriction site at the intracellular side of the PD formed by hydrophobic residues that control sodium conduction. When the VSDs activate, the AG opens, allowing sodium ions to enter the cell. Importantly, activation of the VSDIV is coupled with fast inactivation of the channel (Capes et al., 2013). New work has elucidated this mechanism: initially, the inactivation gate, composed of the Ile-Phe-Met (IFM) motif, is trapped beneath VSDIV in its deactivated state. When VSDIV is activated, the S4 segment moves up and disrupts electrostatic interactions that hold the IFM motif in place, releasing it to bind to the inactivation site in the pore domain (Clairfeuille et al., 2019). This interaction triggers a conformational change that closes the AG and stops sodium flow, placing the channel in a fast-inactivated state. On the extracellular side of the pore lies the selectivity filter (SF), a critical structure that ensures sodium selectivity over other cations, formed by the Asp-Glu-Lys-Ala (DEKA) motif. Conformational changes in the SF have also been implicated in a less understood regulatory process known as slow inactivation (Chen et al., 2024).

Previously, the inability to control membrane voltage during structural determination presented a significant challenge in capturing channels in specific states. Researchers have employed strategies to overcome this limitation, including introducing mutations (Jiang et al., 2021a), using peptide toxins (Clairfeuille et al., 2019), or applying chemical crosslinking (Lee and MacKinnon, 2019) to stabilize ion channels in particular conformational states. Recently, MacKinnon’s lab developed an approach using lipid membrane vesicles with a voltage difference across the membrane to solve cryoEM structures of Eag and KCNQ1 channels in different states (Mandala and MacKinnon, 2022, 2023). Despite this progress, fully resolving the conformational landscape of Na_V_ channel gating and modulation at high resolution remains challenging and hinders the development of subtype-selective, state-dependent Na_V_ channel modulators with therapeutic potential.

Computational methods have become powerful tools to advance our understanding of Na_V_ channel structure, gating, and modulation. The advent of deep learning-based methods for protein structure prediction has significantly enhanced our ability to accurately model protein structures (Pakhrin et al., 2021). Our group demonstrated that AlphaFold2 (Jumper et al., 2021), RoseTTAFold2 (Baek et al., 2021), and ESM Fold (Lin et al., 2023) achieved unprecedented and remarkable accuracy in modeling full Na_V_ channels, with AlphaFold2 outperforming the other methods (Nguyen et al., 2024). AlphaFold2 extends its functionality with AlphaFold Multimer (Evans et al., 2022), designed to predict protein complexes by accurately modeling protein-protein interactions. However, this methodology has yet to be explored for modeling interactions between Na_V_ channels and their protein partners, such as auxiliary subunits or CaM. Additionally, recent studies have shown that AlphaFold2 can model multiple conformational states of proteins by adjusting how the multiple sequence alignment (MSA) is generated and used to generate models (del Alamo et al., 2022; Wayment-Steele et al., 2023; Monteiro da Silva et al., 2024). However, the ability of AlphaFold2 to sample and predict the multiple conformations of Na_V_ channels remains untested.

In this work, we explore AlphaFold2’s capability to sample multiple conformations of Na_V_ channels and assess AlphaFold Multimer’s accuracy in modeling interactions between the Na_V_ α-subunit and key protein partners, including Na_V_ β-subunits and CaM. To enhance conformational variability, we employed a subsampled MSA method (Monteiro da Silva et al., 2024) and evaluated the effects of the number of recycles and the incorporation of state-specific custom templates on conformational sampling. Additionally, we examined how the presence of protein partners influences the conformational landscape of the Na_V_ α-subunit. Our findings demonstrate that AlphaFold2 can accurately model multiple Na_V_ channel states observed in experimental structures and reveal potential intermediate states. Moreover, we show that AlphaFold Multimer accurately models the structure of Na_V_ α-subunit complexes with protein partners and demonstrate that the presence of these partners profoundly reshapes the modeled conformational ensemble of the Na_V_ α-subunit. Our study highlights current advantages and limitations of using deep learning methods to study the dynamic nature of Na_V_ channel structure and expands our understanding of the molecular mechanisms of Na_V_ channel gating and modulation.

## Materials and Methods

### Model generation

All models in this study were generated using colabfold-batch 1.5.0 (localcolabfold) (Mirdita et al., 2022) with a subsampled MSA (Monteiro da Silva et al., 2024). The execution was performed with the following options:

colabfold_batch --num-models 5 --model-type auto --msa-mode mmseqs2_uniref_env \

--num-seeds 20 \

--templates --max-seq 256 --max-extra-seq 512 \

--num-recycle 6 --save-recycles nav17alphafull.fasta outfiles-6r-256-512-sr

The key aspect that makes this colabfold execution a MSA subsampled implementation is the setting of two parameters: --max-seq and --max-extra-seq. The --max-seq parameter defines the maximum number of sequences randomly selected from the original generated master MSA. The target sequence is always included. Then, the remaining sequences are clustered around the selected sequences using Hamming distance. Finally, the cluster centers (the --max-seq sequences chosen originally) are used alongside a sample from each cluster (as defined by the --max-extra-seq parameter) for the structure prediction network.

Previous studies have shown that adjusting these parameters, particularly by reducing their values, can increase the diversity of conformational sampling (del Alamo et al., 2022). In this study, we used values of 256 for --max-seq and 512 for --max-extra-seq, as these settings enhanced conformational diversity without compromising model accuracy in the original study (Monteiro da Silva et al., 2024). A total of 100 models were generated for each study case, using 6 recycles. Intermediate models were saved at each recycle step, resulting in 7 models per generated structure, ranging from recycle 0 to recycle 6, making a total of 700 models per test case (see Data Availability).

### Model Analysis

All models were visually analyzed using UCSF ChimeraX (Goddard et al., 2018). Distances were automatically calculated with custom Python scripts utilizing the PyRosetta package (Chaudhury et al., 2010). All other analyses, calculations, and figure generation were conducted with custom Python scripts. Pore analysis of the channels was conducted using the MOLEonline web interface (Pravda et al., 2018).

## Results

### Defining distance coordinates to identify channel states

**Figure 1.**
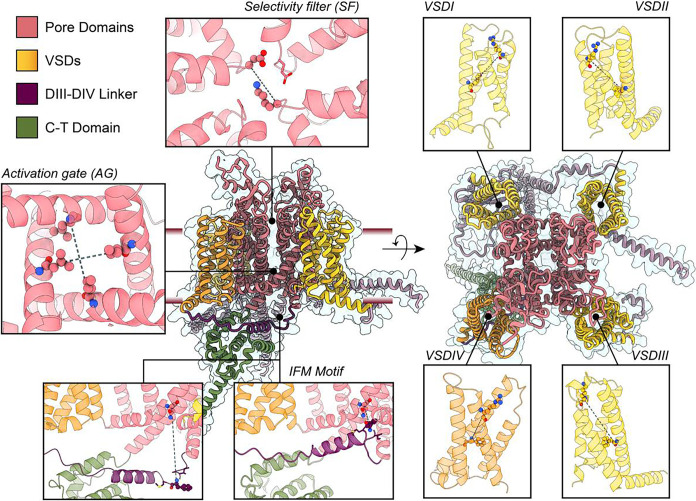
Selected distances to use as coordinates to identify channel states. We focused on seven different regions of Na_V_ channels with known conformational dynamics: the four VSDs, the SF, the AG and the IFM motif. The calculated distances are illustrated in the figure with dashed lines.

Na_V_ channels are complex molecular machines that exhibit dynamic transitions between distinct conformational states, influenced by voltage, regulatory protein partners, ion concentrations, lipids, and other factors like phosphorylation and glycosylation. To evaluate AlphaFold2’s ability to sample this conformational diversity, we focused on key regions of the Na_V_ α-subunit whose structural dynamics have been extensively characterized (Fig. 1). We defined specific distance coordinates for each of these Na_V_ α-subunit regions that could be calculated across all generated models to assess different conformational states (Table S1).

For the voltage-sensing domains (VSDs I-IV), we measured the distance between the α-carbon of the first gating charge in the S4 segment (GC1-S4) and the α-carbon of the residue forming the hydrophobic constriction site in the S2 segment (HC-S2). Larger values of this distance (GC1-S4 – HC-S2) indicate an activated or “up” VSD state, while smaller values would represent a deactivated or “down” VSD state. In the activation gate (AG), we calculated the distances between the α-carbons of opposing residues that form the hydrophobic intracellular gate of the channel (named AG1 and AG2, for S6_I_-S6_III_ and S6_II_-S6_IV_ respectively). This allows us to distinguish whether the activation gate is open or closed. We also measured the distance between the α-carbon of the phenylalanine in the IFM motif and the α-carbon of the aspartic acid within the IFM binding site in the pore domain (IFM – PD distance). A low value for this distance indicates that the IFM motif is bound, placing the channel in a fast-inactivated state. Additionally, we assessed the distance between the α-carbons of the lysine and aspartic acid residues in the DEKA motif of the selectivity filter (SF), naming this distance SF-D – K. Variations in this distance indicate changes in the dilation of the SF, which may represent different conformational states associated with slow inactivation (Vilin and Ruben, 2001). To assess whether the generated models encompass the range of experimentally observed conformations, we calculated these same distances in available structures of human Na_V_ channels, as well as specific non-human Na_V_ channels representing distinct states, for comparison (Table S2).

After defining these distance coordinates across all human Na_V_ channels, we proceeded with the modeling using ColabFold (Mirdita et al., 2022). We generated 100 models per full channel using a subsampled MSA with six recycles, saving intermediate recycles for analysis (see Methods). Note that “recycles” in AlphaFold modeling refer to iterative steps of feeding the predicted structure back into the neural network to refine and improve the model’s accuracy. Although we initially analyzed each region of interest separately, the models included the full channel sequence, enabling us to subsequently evaluate the coupling between the states of different regions. We modeled all human voltage-gated Na_V_ channels (hNa_V_1.1 to hNa_V_1.9) and the non-voltage-gated hNa_X_ channel with a Na_V_-like architecture (Noland et al., 2022). Modeling hNa_X_ allowed us to evaluate whether the state distributions of AlphaFold models of hNa_V_ channels versus the hNa_X_ channel reflected unique structural and functional features of the Na_X_ channel. In addition to calculating the distance coordinates described above, we evaluated the predicted local distance difference test (pLDDT) values for all models as the confidence metric. Generally, pLDDT values above 90 indicate very high confidence, 70–90 indicate good confidence, 50–70 indicate low confidence, and below 50 indicate very low confidence. While AlphaFold provides a global pLDDT score as a measure of overall model confidence, since this is a metric reported at the residue backbone level, we focused on subset pLDDT values for specific regions of interest: the VSDs, the four residues comprising the AG, the DEKA motif in the SF, and the three residues forming the IFM motif. Furthermore, we calculated an adjusted-global pLDDT score that excluded the large unstructured intracellular loops. These regions typically receive low and variable pLDDT values, which can distort the overall score and lead to misleading variations in the global pLDDT. By focusing only on well-structured regions of the channel, the adjusted score provides a more accurate reflection of the model’s confidence.

### AlphaFold samples multiple VSD states with varying degrees of gating charge translocations

**Figure 2.**
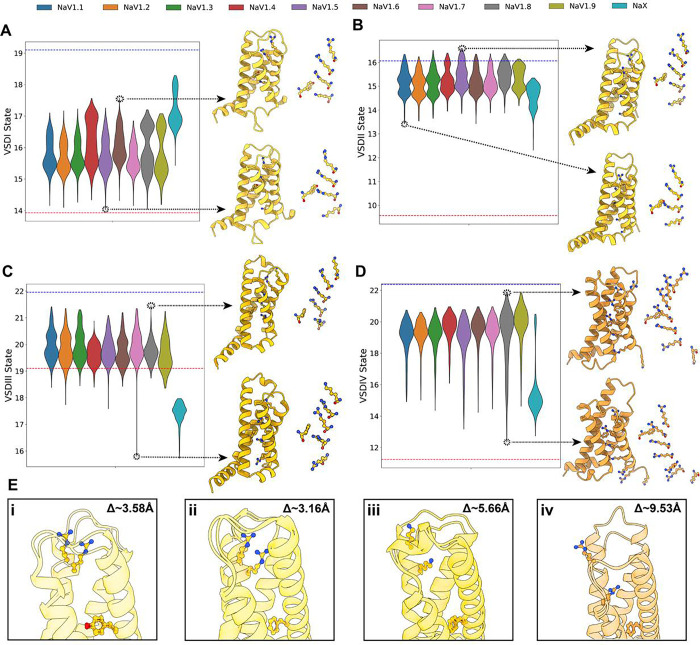
Distribution of VSD states in generated AlphaFold models of hNa_V_ channels. (A-D) Distribution of the GC1-S4 – HC-S2 distances for the four VSDs across the nine hNa_V_ channel subtypes plus the hNa_X_ channel. Blue dashed lines mark the largest distance observed in experimental structures for that coordinate, while the red dashed lines represent the lowest; this provides an idea of the observed range of states in experimental structures. The VSD models with the largest and lowest distance coordinates for each of the VSDs are shown highlighting the relative positioning of the gating charges in relation to the hydrophobic constriction site. (E) Superimposition of the most activated and deactivated models for each of the VSDs shown in panels A-D, highlighting the difference in the distance coordinates between the most activated and deactivated models.

We first focused on the states of the VSDs (Fig. 2). Overall, we observed that various conformational states were sampled, with the extent of this sampling varying across VSDs. Notably, within each VSD, the state distribution was consistent across hNa_V_ subtypes, meaning that for a given VSD, the sampled states were similar among the different hNa_V_ subtypes. The remarkable exception was hNa_X_, which is not voltage-dependent, and we expected to observe a distinct state distribution. Indeed, hNa_X_’s state distributions stand out across all four VSDs, aligning with the fact that the VSD-like domains of Na_X_ are not able to sense voltage and adopt different conformational states compared to Na_V_ channels. Below, we analyze each VSD in detail.

In the VSDI, we observed bimodal distributions of the GC1-S4 – HC-S2 distances, which fall within the range of conformational states seen in experimental structures (Fig. 2 A). The most activated state was observed in the experimental structure of hNa_V_1.3 (PDB: 7W7F) (Li et al., 2022) with a distance of 19.1 Å, while the most deactivated state was seen in hNa_V_1.8 (PDB: 7WFR) (Huang et al., 2022b) with a distance of 14.3 Å. In our models, the lowest distance was found in a model of hNa_V_1.5 at 14.0 Å, and the upper limit in a model of hNa_V_1.6 with a distance of 17.6 Å. Visual comparison of these models, specifically the relative positions of the gating charges, indicates that these structures represent two distinct VSDI states: an activated state has three of the four gating charges in the S4 segment positioned above the hydrophobic constriction site in the S2 segment, while a partially deactivated state shows only two gating charges above this site, indicating a shift of one “click” downward (Fig. 2 E, panel i; Video S1). Interestingly, hNa_X_ VSDI samples larger GC1-S4 – HC-S2 distances, reaching up to 18.3 Å, and reflecting further activated states of VSDI S4. Notably, Na_X_ VSDI contains only three positively charged residues in S4 compared to four positively charged residues in Na_V_ VSDIV (Noland et al., 2022).

For VSDII GC1-S4 – HC-S2 distances, we observed activated and partially deactivated states, differing by one “click” of gating charge movement, with three versus two gating charges in the S4 segment positioned above the hydrophobic constriction site in the S2 segment (Fig. 2 B). Experimental structures exist for deactivated conformations of VSDII, such as chimeric Na_V_Ab-hNa_V_1.7-VSDII stabilized by the peptide toxin Huwentoxin-IV (PDB: 7K48) (Wisedchaisri et al., 2020), which shows a distance of 9.6 Å and only one gating charge above the hydrophobic constriction site. However, none of our human Na_V_ models sampled these fully deactivated conformations of the VSDII, with the lowest distance observed in a model of hNa_V_1.1 at 13.4 Å. hNa_X_ VSDII stands out, sampling lower distances up to 12.3 Å. Notably, hNa_X_ VSDII contains the same number of positively charged residues in S4 (five) as in Na_V_ VSDII S4 (Noland et al., 2022).

We observed larger GC1-S4 – HC-S2 distances for VSDIII (Fig. 2 C), as this VSD contains an additional gating charge in the S4 segment. Our models sampled the observed conformational space from experimental structures, with hNa_V_1.1 (PDB: 7DTD) (Pan et al., 2021) marking the upper limit at 21.8 Å and hNa_V_1.5 (PDB: 7DTC) (Li et al., 2021b) marking the lower limit at 20.9 Å. This highlights the limited observable states for Na_V_ VSDIII in experimental structures, where in all of them, four gating charges are positioned above the hydrophobic constriction site. In contrast, our models showed a broader range of states. The most activated model generated was for hNa_V_1.8 with a distance of 21.5 Å and four gating charges positioned above the hydrophobic constriction site. The most deactivated VSDIII was found in a model of hNa_V_1.7 with a distance of 15.8 Å, below the limit observed in experimental structures, with three gating charges positioned above the constriction site, corresponding to a shift of one “click” downward (Fig. 2 E, panel iii; Video S1). This state has not yet been observed in any experimental structure of hNa_V_ VSDIII. Additionally, hNa_X_ VSDIII stood out with significantly lower GC1-S4 – HC-S2 distances, consistent with its experimental structure, which also displayed a lower distance of ~18 Å and reflected further deactivated states of S4. Notably, hNa_X_ VSDIII contains only four positively charged residues in S4 compared to five positively charged residues in Na_V_ VSDIII (Noland et al., 2022).

VSDIV (Fig. 2 D) exhibited the broadest range of sampled GC1-S4 – HC-S2 distances. We observed two “clicks” of difference in the translocation of gating charges across the hydrophobic constriction site. The largest and smallest distances were observed in hNa_V_1.8 models, where the most activated state had four gating charges above the constriction site and the most deactivated state had two, resulting in a distance difference of 9.5 Å (Fig. 2 E, panel iv; Video S1). These results encompass the range of states observed in experimental structures, where the upper limit is marked by hNa_V_1.1 (PDB: 7DTD) (Pan et al., 2021) with 22.4 Å, and the lower limit by the Na_V_PaS-hNa_V_1.7 chimera, which has VSDIV trapped in a deactivated state by an α-scorpion toxin (PDB: 6NT4) (Clairfeuille et al., 2019) with a distance of 11.2 Å. The deactivated VSDIV in this chimera shows two gating charges above the hydrophobic constriction site, similar to what we observed in the hNa_V_1.8 model. hNa_X_ VSDIV models also sampled the GC1-S4 – HC-S2 states resulting from the two ‘click’ translocations. However, for hNa_X_, the most frequently sampled state was the more deactivated state, in contrast to Na_V_ channels, where the most frequently sampled state was the activated one. Notably, hNa_X_ VSDIV contains only three positively charged residues in S4 compared to six positively charged residues in Na_V_ VSDIV (Noland et al., 2022).

Overall, these results demonstrate AlphaFold2’s ability to partially capture the conformational diversity of Na_V_ channel VSDs. By analyzing the distribution of GC1-S4 – HC-S2 distance coordinates across VSDs and comparing them to experimental structures, we have identified different potential conformational states in the VSDs of all hNa_V_ channels. Notably, AlphaFold2 is able to generate models that sample intermediate states between fully activated and deactivated conformations. These intermediate states may represent important steps in the activation and deactivation pathways. However, our results also reveal certain limitations. For VSDI, VSDII, and VSDIII, we observed only one “click” of activation across the sampled models, potentially missing more deactivated states, particularly for VSDII, where more deactivated conformations have been observed in experimental structures (Wisedchaisri et al., 2020). Nevertheless, the ability of AlphaFold2 to generate diverse VSD conformations, including intermediate states, highlights its potential to provide deeper insights into the structural transitions involved in VSD activation and deactivation, which may aid in refining models of channel gating and modulation mechanisms.

### AlphaFold modeling reveals frequent sampling of IFM motif in bound and unbound states, along with potential slow inactivation conformations of the selectivity filter

**Figure 3.**
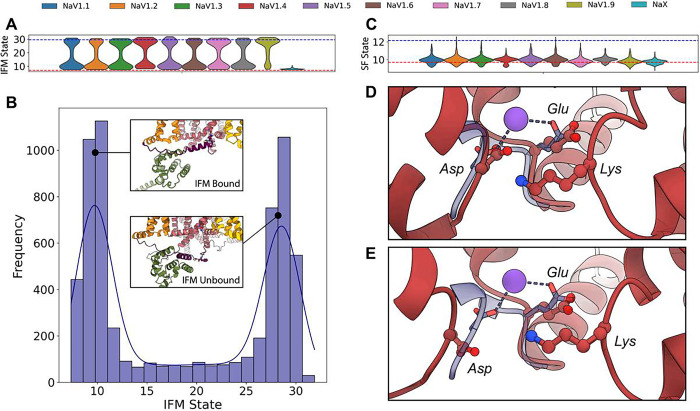
Distribution of IFM and SF states in generated AlphaFold models of hNa_V_ channels. (A) Distribution of the IFM – PD distance for the nine hNa_V_ channels plus hNa_X_. (B) Overall distribution of IFM-PD distances in all generated models. (C) Distribution of the SF-D – K distance for the nine hNa_V_ channels plus hNa_X_. (D, E) Models representing the generated state with lowest (D, 9 Å) and largest (E, 12.6 Å) SF-D – K distances. The side chains of the aspartate, glutamate and lysine of the DEKA motif are shown. The superimposed SF of the experimental structure of hNa_V_1.4 (PDB: 6AGF) (Pan et al., 2018) is shown in purple to illustrate the structural arrangement of the SF that allows Na^+^ coordination. For A and C, the blue dashed lines mark the largest distance observed in experimental structures for that coordinate, while the red dashed lines represent the lowest.

When analyzing the distribution of IFM states using the IFM – PD distance, we observe a distinct bimodal pattern: one state where the IFM is bound to the pore domain, corresponding to a fast-inactivated channel with an IFM – PD distance of ~8 Å, and another state where the C-terminal region traps the IFM, unbound from the pore domain, with the IFM positioned nearly 30 Å away from its binding site in the pore. Interestingly, all nine hNa_V_ channels sampled both states (Fig. 3 A), although with varying frequencies. For example, hNa_V_1.2 shows a higher occurrence of the bound state, while hNa_V_1.9 samples the unbound state more frequently. Once again, hNa_X_ stands as an outlier, with only the bound state being sampled. When we analyzed the global distribution of IFM states across the nine hNa_V_ channels (Fig. 3 B), we found that, despite the IFM motif predominantly being observed in the bound state in experimental structures (Table S2), both the bound and unbound states were frequently sampled in our models. A notable result is the appearance of intermediate IFM states between the bound and unbound conformations—states that have not yet been observed in experimental structures.

In the SF region, we focused on identifying potential SF conformations that might represent slow-inactivated states. Most experimental structures show the SF-D – K distance ranging from 9.7 to 11.2 Å, corresponding to conductive states of the SF (Table S2). However, a recent experimental structure with a putative slow-inactivated state showed a significantly larger distance of 12.2 Å, accompanied by clear structural changes that suggest a non-conductive, slow inactivated conformation (Chen et al., 2024). Interestingly, the state distribution across the nine hNa_V_ channels indicates that, while the state ~10 Å is the most frequently sampled (Fig. 3 C), some channels show low-frequency sampled models with SF-D – K distances similar to the putative slow-inactivated state ~12 Å. Visually comparing models with the smallest and largest SF-D – K distances, we observe that the model with the lowest distance (9 Å), corresponding to hNa_V_1.9, shows the aspartate and glutamate residues of the DEKA motif positioned to allow sodium coordination (Fig. 3 D). More intriguingly, in the model with the largest distance (12.6 Å) corresponding to hNa_V_1.2, the aspartate is further from the glutamate, disrupting the spatial arrangement necessary for sodium coordination, suggesting that this model may represent a slow-inactivated, non-conductive state (Fig. 3 E; Video S2). This is particularly significant, as such a state has not been observed in any experimental structure of a SF containing the DEKA motif. The previously mentioned experimental structure of a putative slow-inactivated Na_V_ channel corresponds to an ancient eukaryotic symmetric channel, Na_V_Eh, that retains the EEEE motif in the SF. Notably, the frequency of sampling of this potential slow-inactivated state varied across the hNa_V_ family models. Channels hNa_V_1.2 and hNa_V_1.6 had the highest number of models in this state, with 8 and 5 models respectively showing SF-D – K distances greater than 11.5 Å. In contrast, hNa_V_1.4, hNa_V_1.8, and hNa_V_1.9 did not produce models with SF-D – K distances corresponding to this state, with the maximum sampled distance being around 11.3 Å (Fig. 3 C). Future studies using more advanced tools such as AlphaFold3 (Abramson et al., 2024), that support modeling with sodium ions explicitly, will be necessary for a more accurate assessment of SF conformational dynamics under physiological conditions.

### AlphaFold predicts known and novel activation gate states

To investigate the conformational states of the AG across the hNa_V_ channels, we focused on the area formed by multiplying the two calculated distances, AG1 and AG2, which provide insight into the backbone-determined state of the gate. While we acknowledge that the actual space available for ion permeation depends on the positioning of side chains within the gate, our focus here is on the overall structural state dictated by the backbone. When we calculated the equivalent area in available experimental structures of human channels, we observed that the lower limit was 151 Å^2^, found in hNa_V_1.7 in complex with the peptide toxin Huwentoxin-IV (PDB: 7W9P) (Huang et al., 2022a), a structure believed to represent a closed channel state. The experimental structure of the cockroach Na_V_PaS (PDB: 5X0M) (Shen et al., 2017) exhibits an even more tightly closed pore, with an area of 129.6 Å^2^. On the other hand, experimental structures hypothesized to represent open states showed areas ~200 Å^2^, such as the rat Na_V_1.5/QQQ mutant (PDB: 7FBS) (Jiang et al., 2021a).

**Figure 4.**
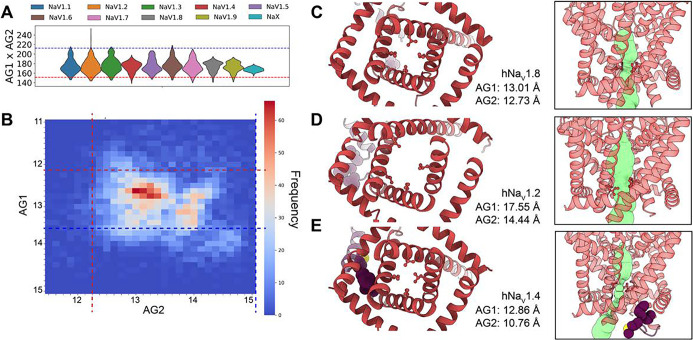
Distribution of AG states in generated AlphaFold models of hNa_V_ channels. (A) Distribution of the AG1xAG2 area for the nine hNa_V_ channels plus hNa_X_. (B) Frequency heatmap showing the combined frequency of the AG1 and AG2 coordinate distances in all hNa_V_ models; the blue dashed lines mark the largest distance observed in experimental structures and the red dashed lines represent the lowest. (C-E) Models representing the most frequent (C), largest (D) and lowest (E) AG area sampled in our models; the IFM motif is highlighted in purple, and the calculated volume of channel pore is represented with the green surface.

Our models of the nine hNa_V_ channels revealed a distribution of activation gate areas that encompasses this experimental range (Fig. 4 A). The smallest area was observed in a model of hNa_V_1.4 at 138.3 Å^2^, while the largest, an outlier, was a model of hNa_V_1.2 with an area of 253 Å^2^. This outlier model of hNa_V_1.2 was notable, as no other hNa_V_ channel sampled such a large area. Most channels, however, sampled states with areas larger than 200 Å^2^, aligning with structures believed to represent open states, except for hNa_V_1.4, hNa_V_1.8, and hNa_V_1.9, which showed smaller areas. hNa_X_ exhibited a narrower distribution compared to the other channels.

When we analyzed the AG1 and AG2 distances together (Fig. 4 B), the most frequently sampled state had AG1 distances between 12.6–12.8 Å and AG2 distances between 13–13.1 Å, resulting in an area range of 163.8–167.3 Å^2^ which represents either a closed or inactivated state. To gain more detailed insights, we visually analyzed models presenting the smallest and largest activation gate areas, as well as a model corresponding to the most frequently sampled state (Video S3). In the model with the most frequently sampled area (Fig. 4C), we observed a narrow activation gate and an unbound IFM motif, which we identified as a possible closed state. The model with the largest area, the outlier hNa_V_1.2 mentioned earlier (Fig. 4 D), showed a dramatic displacement of the DI-S6 segment, creating a clear open pathway. This model also exhibited the IFM motif in an intermediate state between bound and unbound, suggesting that it may represent a low-frequency or short-lived open state. Finally, the model with the smallest area, found in hNa_V_1.4 (Fig. 4 E), displayed a fully bound IFM motif and a closed pathway, which we identified as an inactivated state.

Although this may be an oversimplification—given the likely existence of multiple distinct open, closed, and inactivated states—the key finding is that AlphaFold successfully sampled a wide range of activation gate conformations across all hNa_V_ channels, from putative closed and inactivated to fully open states. Notably, this includes states that have not yet been experimentally observed in most of hNa_V_ channel structures.

### AlphaFold models reveal region-specific relationships between conformational states and model confidence

After demonstrating that AlphaFold2 can model multiple states of Na_V_ channels, we asked two key questions: (1) Do specific states receive distinct pLDDT values, indicating varying model confidence? (2) How does the number of recycles aVect the distribution of these states This section addresses these questions by analyzing model confidence, represented by pLDDT values, and state distributions across recycles. For the pLDDT values, we used a subsetting pLDDT calculation, reporting the average pLDDT of the residues forming the region of the channel of interest. All models in this study were generated using six recycles and saving intermediate recycle models.

**Figure 5.**
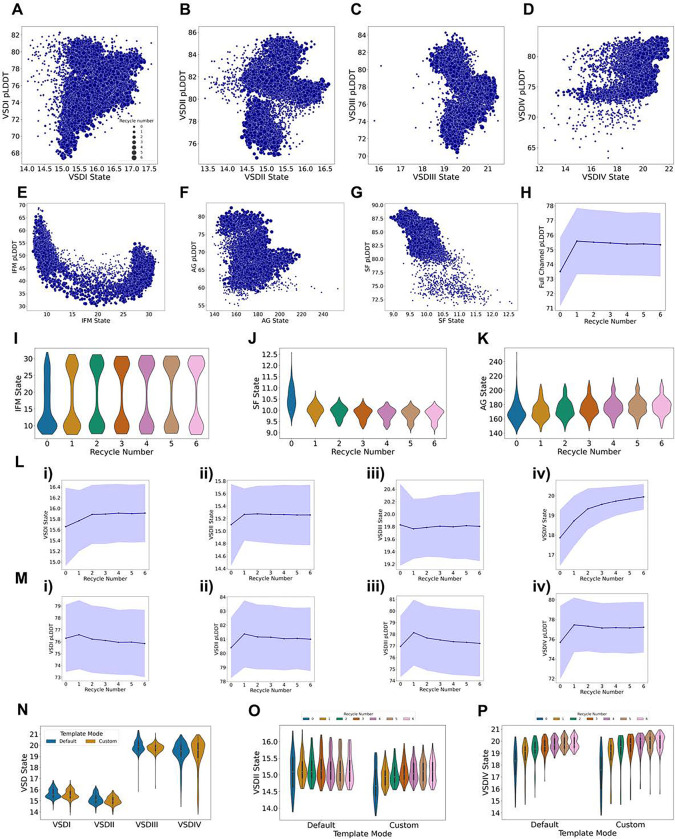
Relationships between state distributions, model pLDDT, number of recycles and use of custom templates. (A-G) Relationship between the state distributions of the seven investigated regions and the corresponding pLDDT values of these regions; dot sizes represent the recycle number. (H) Evolution of the average adjusted-global pLDDT values across recycles; shaded outlines represent standard deviation. (I-L) Evolution of state distributions across recycles for the studied regions. (M) Evolution of average pLDDT values of the four VSDs across recycles; shaded areas indicate the standard deviation. (N) Comparison of the distribution of hNa_V_1.7 VSD states with the default and custom template modes. (O,P) Comparison of the distribution of hNa_V_1.7 VSDII (O) and VSDIV (P) states across recycles with the default and custom template modes.

We first examine the correlation between the distinct conformational states of key regions in the Na_V_ channels and their respective pLDDT values. For the VSDs, we observe distinct distributions for each of the four domains. In VSDI (Fig. 5 A), both deactivated and activated states achieve high confidence; however, a trend emerges where more activated states tend to receive higher pLDDT scores. In VSDII (Fig. 5 B), the most confident models are generally those in intermediate states with pLDDT values up to 86, while the more activated conformations have pLDDT values ~82. A similar pattern is seen in VSDIII (Fig. 5 C), whereas in VSDIV (Fig. 5 D), we note that more deactivated states have slightly lower confidence scores (pLDDT ~75), while fully activated states exceed a pLDDT of 80.

For the IFM Motif (Fig. 5 E), a clear correlation emerges: the bound state consistently achieves the highest pLDDT values (approaching 70), while the unbound state—where the IFM motif is trapped by the C-T domain—shows moderate values (~55). Intermediate states between the bound and unbound conformations exhibit the lowest pLDDT values. Analyzing the AG (Fig. 5 F), the highest pLDDT scores (~80) are associated with closed or inactivated states (area between 150–200 Å^2^). Possible open states with activation gate areas around ~200–220 Å^2^ cluster around pLDDT values of 65–70, whereas outlier models with activation gate areas larger than 220 receive lower pLDDT values, ~60. The selectivity filter (Fig. 5 G) also exhibits a trend: less dilated conformations have higher pLDDT scores (reaching 90), whereas states showing greater dilation have reduced model confidence (~70–75).

These observations reveal that the relationship between conformational states and pLDDT generates distinct distributions for each Na_V_ region, providing insight into how model confidence varies across states. When further analyzing the relationship between states and the number of recycles, a pattern emerges in which outlier states, such as extreme conformations, predominantly appear at Recycle 0, with models clustering more tightly as the number of Recycles increases (Fig. 5 A–G). This trend leads us to investigate the evolution of state distributions as a function of the number of recycles.

Before analyzing the impact of recycles on the distribution of specific states, we first examined how recycling affects the overall confidence of the models. Fig. 5 H shows that the adjusted-global pLDDT score for the full Na_V_ channel increases from recycle 0 to recycle 1 on average, after which it stabilizes. This suggests that total model confidence improves after initial recycling but plateaus thereafter.

In examining the distribution of states over an increasing number of recycles, Fig. 5 I shows that intermediate IFM states are primarily sampled at recycle 0, while increasing recycles tend to favor either the bound or unbound IFM state. A similar trend is observed for the selectivity filter (Fig. 5 J), where more dilated conformations are sampled at recycle 0. For the activation gate (Fig. 5 K), we observe that super-open states are sampled exclusively at recycle 0, while recycles 1 through 6 converge on states consistent with experimental structures (between 150 and 200 Å^2^ in gate area). The VSDs exhibit a notable trend: deactivated states are more commonly sampled at recycle 0, shifting toward more activated states in subsequent recycles, particularly in VSDIV (Fig. 5 L). VSDIII appears to have limited diversity in sampled states, consistent with its limited observed diversity in experimental structures. Fig. 5 M highlights the evolution of pLDDT values for the VSDs across recycles, showing a slight increase from recycle 0 to 1, followed by stabilization.

Our results reveal a relationship between pLDDT and the conformational states that depends on the specific region of the channel with distinct patterns emerging. While some models with outlier states may have slightly lower pLDDT scores, the lowest values remain around a pLDDT of 60–70, which suggests moderate confidence, meaning the reliability of these models cannot be completely dismissed. The number of recycles significantly impacts the generated conformational distribution, with recycle 0 showing the greatest diversity of states, while increased recycling tends to bias the modeling towards particular states in each region.

### Minimal impact of state-specific custom templates on VSD state distributions

After evaluating the effects of recycle number and the relationship between confidence and conformational states, we wanted to assess whether using state-specific custom templates could bias AlphaFold towards states represented by those in the experimental structure templates. As a practical case, we focused on the Na_V_1.7 channel, which has several structures of the VSDII in the deactivated conformation. To conduct this analysis, we used two specific templates: PDB 6N4R (Xu et al., 2019) and PDB 7K48 (Wisedchaisri et al., 2020). These structures are chimeric constructs in which the human Na_V_1.7 VSDII sequence is grafted onto a bacterial channel Na_V_Ab. Both structures are four-fold symmetric, with all four VSDs in the deactivated conformation, stabilized by peptide toxins and this symmetry provides four copies of the deactivated VSD per structure.

We generated the same number of models under identical conditions for hNa_V_1.7, but instead of using the default template mode (where AlphaFold automatically selects relevant templates from a predefined refined version of the PDB to guide predictions), we used a custom template mode, providing only the two aforementioned structures as templates. The results indicate that the overall effect of using these templates is minimal. When observing the distribution of states across the four VSDs, we found no significant differences between models generated with the default template mode and those generated with the custom template mode, particularly in VSDII, where an effect would be most expected (Fig. 5 I).

To investigate whether the template mode might have a more pronounced effect at lower recycle numbers, we examined the VSD state distribution from recycle 0 to 6 in the default and custom template model sets. In VSDII, we observed that at recycle 0 and 1, more deactivated states were sampled with the custom templates compared to the default mode (Fig. 5 J). However, as the number of recycles increased, the differences between default and custom template modes diminished. Interestingly, a more pronounced effect was observed in VSDIV (Fig. 5 K) as using the custom template mode resulted in more deactivated states being sampled. Specifically, with the default mode, no deactivated states were sampled in recycles 4, 5, and 6, whereas with the custom template mode, deactivated states continued to be sampled even at these higher recycles.

Overall, our findings suggest that using custom templates can introduce a slight bias in state sampling, particularly at lower recycle numbers. However, as the number of recycles increases, this effect tends to diminish, resulting in similar distributions regardless of the template mode used.

### Correlations between predicted conformational states of different Na_V_ channel regions

The next question we asked was whether the states of different channel regions correlate with each other. This question is related to the known molecular mechanisms underlying the function of Na_V_ channels.

**Figure 6.**
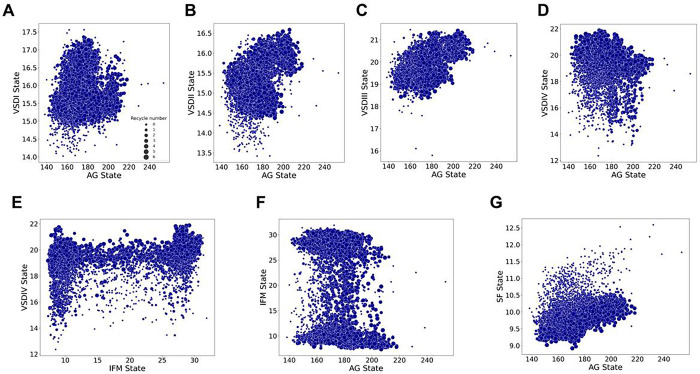
Correlations between conformational states of different Na_V_ channel regions. (A-D) Relationships between the AG state and the degree of activation of the four VSDs in all generated hNa_V_ models. (E) Relationships between the IFM motif state and the degree of activation of the VSDIV. (F) Relationships between the AG and IFM motif states. (G) Relationships between the AG and SF states. In all plots, the dot size represents recycle number.

Firstly, we investigated whether the state of each of the four VSDs correlates with the degree of opening or closing of the AG. The activation of the Na_V_ VSDI, VSDII, and VSDIII generates the mechanical work needed to open or close the pore or AG (Chanda and Bezanilla, 2002; Muroi et al., 2010). As a result, we would expect activated Na_V_ VSDI, VSDI, and VSDIII states to be associated with more open AGs. Experimental measurements of conformational changes within Na_V_ VSDs revealed that VSDIV changes conformation after the other three VSDs, with a time course that follows fast inactivation and suggests that the VSDIV in the up-state conformation is sufficient for fast inactivation to occur (Capes et al., 2013). We examined the correlation between each of the GC1-S4 – HC-S2 distances defining the four VSD states and the area of the AG (Fig. 6 A–D). For VSDI and VSDIV, we observed a nuanced relationship: while intermediate activation states of these VSDs (GC1-S4 - HC-S2 distances ~15 Å for VSDI and 19 Å for VSDIV) correlated with wide AG area distributions (ranging from 140 to more than 200 Å^2^), more activated states were associated with narrower AG area distributions (140–180 Å^2^). On the other hand, for VSDII and VSDIII, we observe a clearer trend: as these VSDs become more activated, larger AG areas (more than 200 Å^2^) are more frequently sampled, indicating a correlation between open AG states and more activated VSDII and VSDIII.

The next question we addressed was the relationship between the state of the IFM motif and VSDIV. From previously hypothesized molecular mechanisms on Na_V_ fast inactivation (Capes et al., 2013; Clairfeuille et al., 2019), we would expect that when VSDIV is deactivated, the IFM motif remains unbound and is retained below the VSDIV by the C-T domain, while activation of VSDIV releases the IFM motif to bind to the pore domain. However, AlphaFold2 generated models with VSDIVs in both deactivated and activated states with the IFM motif either bound or unbound (Fig. 6 E). Notably, when the IFM motif is in the bound state (low IFM – PD distances) we see a wider distribution of frequently sampled VSDIV states, while when it is unbound, we see a frequently sampled VSDIV state distribution shifted to more activated states. This finding was contrary to our initial expectations based on known molecular mechanisms. Interestingly, intermediate states of the IFM motif, where it is partially bound or unbound, tend to occur at a specific activation level of VSDIV.

We also examined the relationship between the AG opening and the IFM motif. We would expect that when the IFM motif is bound, the AG area would be smaller, as the IFM motif stabilizes the AG in closed states, which occlude the permeation path. However, we found no consistent relationship (Fig. 6 F); all combinations occur, and, in fact, the most open states (areas larger than 200 Å^2^) occur more frequently with the IFM motif bound, though these states do appear with both bound and unbound IFM motif states. Additionally, we analyzed the relationship between the AG and the state of the SF (Fig. 6 G). We observed that when the AG presents areas between 180 and 200 Å^2^, there seems to be a convergence of the SF dilation state ~10 Å.

Overall, these findings indicate that the relationships between the Na_V_ states of different channel regions are generally diffuse in the generated AlphaFold2 models, with a wide variety of state combinations observed. Remarkably, we observe an apparent coupling between the VSDII and VSIII activation and AG opening. However, drawing specific conclusions from AlphaFold2 ensembles of models is challenging and requires further analysis of relative frequencies and a more in-depth investigation into these relationships.

### High accuracy of α and β-subunit complex models generated by AlphaFold Multimer agree with experimental structures

Up until now, we have focused on modeling the Na_V_ α-subunit alone, which forms the core of the channel itself. However, these channels are regulated by auxiliary protein partners, the most physiologically significant of which are the auxiliary β-subunits: β1, β2, β3, and β4 (Namadurai et al., 2015). These β-subunits have two domains: a transmembrane domain and an extracellular immunoglobulin domain. We modeled hNa_V_1.7 paired with each of the four β-subunits generating 100 models with 6 recycles per case. Additionally, we used hNa_V_1.1 as a control to validate the replicability of our results. For each of these cases, we wanted to assess whether the generated models align with the experimental structures available for the different complexes.

**Figure 7.**
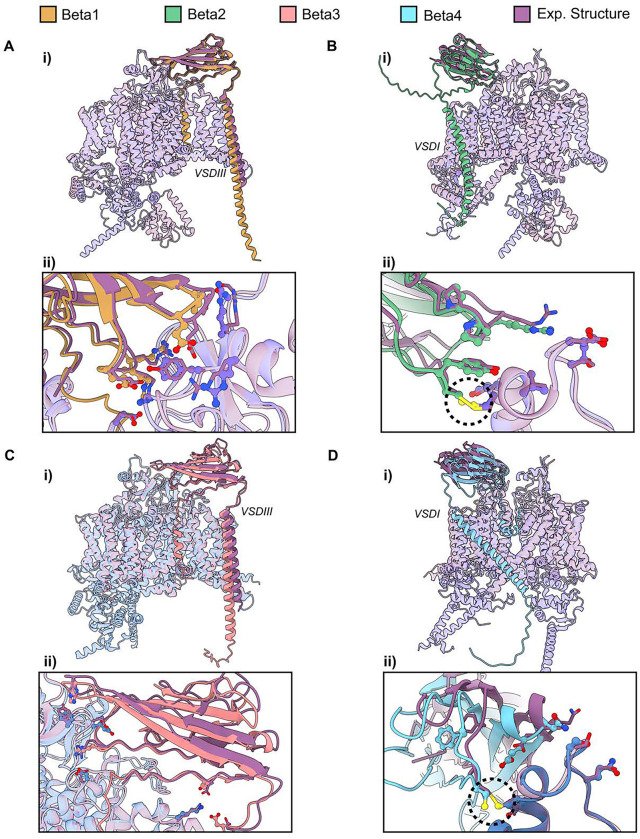
Comparison of top models of hNa_V_1.7 in complex with auxiliary β-subunits with reference experimental structures. (A-D, i) Superimposition of top ranked AlphaFold models of hNa_V_1.7 in complex with the four auxiliary β-subunits with the corresponding experimental structure of reference: (A,B) hNa_V_1.7-β1-β2 complex, PDB: 7W9K (Huang et al., 2022a); (C) hNa_X_- β3 complex, PDB: 7TJ8 (Noland et al., 2022); (D) hNa_V_1.1-β4 complex, PDB: 7DTD (Pan et al., 2021). (A-D,ii) Detailed view of the α-β interface comparing top models (side chains represented as balls and sticks) with experimental structures (side chains shown as sticks).

We compared the top-ranked model of hNa_V_1.7 paired with each of the β-subunits to reference experimental structures (Fig. 7 A–D, i). Overall, we observed an almost perfect alignment between the experimental structures and our models. For β1 (Fig. 7 A), the β-subunit is positioned next to VSDIII, consistent with the reference structure (PDB: 7W9K, hNa_V_1.7-β1-β2 complex) (Huang et al., 2022a), with an RMSD value of 0.7 Å when superimposing the auxiliary subunits. For β2 (Fig. 7 B), the immunoglobulin domain aligns perfectly with the experimental structure (PDB: 7W9K, hNa_V_1.7-β1-β2 complex) (Huang et al., 2022a) and is situated above VSDI, with an RMSD value of 0.4 Å. For β3 (Fig. 7 C), both the immunoglobulin domain and transmembrane domain align well with the reference experimental structure (PDB: 7TJ8, hNa_X_-β3 complex) (Noland et al., 2022) and are positioned next to VSDIII, with an RMSD value of 2.2 Å. For β4 (Fig. 7 D), while we observe some shift relative to the reference experimental structure (PDB: 7DTD, hNa_V_1.1-β4 complex) (Pan et al., 2021) when superimposing the full channel, it is also accurately positioned next to VSDI, with an RMSD value of 1.2 Å when superimposing the β4 subunit alone. The highest RMSD value is for β3, which would be expected as the comparison is with the reference experimental structure of the Na_X_ channel, which is not a Na_V_ channel. Therefore, the observed difference in RMSD could reflect conformational changes specific to the β-subunit that depend on the type of α-subunit involved. For both β2 and β4, while the transmembrane domain is known to exist, it is not resolved in the available experimental structures, likely due to conformational flexibility. Despite this, the immunoglobulin domain aligns well, and the transmembrane domain appears slightly tilted relative to the membrane axis in our models. Top models of hNa_V_1.1 in complex with the β-subunits showed similar patterns and also aligned well with the reference experimental structures (Fig. S1).

An important distinction is that β2 and β4 interact with the Na_V_ α-subunit by forming disulfide bridges, whereas β1 and β3 interact with the Na_V_ α-subunit non-covalently. When examining the atomic details of the interface for all four β-subunits (Fig. 7 A–D, ii), we found nearly perfect alignment between the experimental structures and our models, both in the interactions present and in the conformations of the side chains of amino acids at the interface. Notably, for β2 and β4, the cysteines responsible for forming disulfide bridges are positioned accurately to establish these bonds, with the sulfur atoms of the cysteines nearly perfectly aligned with the experimental structures (Fig. 7 B and D, panel ii, dashed circles). Although AlphaFold does not model covalent bonds between different protein partners, the positioning of the sulfur atoms suggests a high likelihood of disulfide bridge formation.

**Figure 8.**
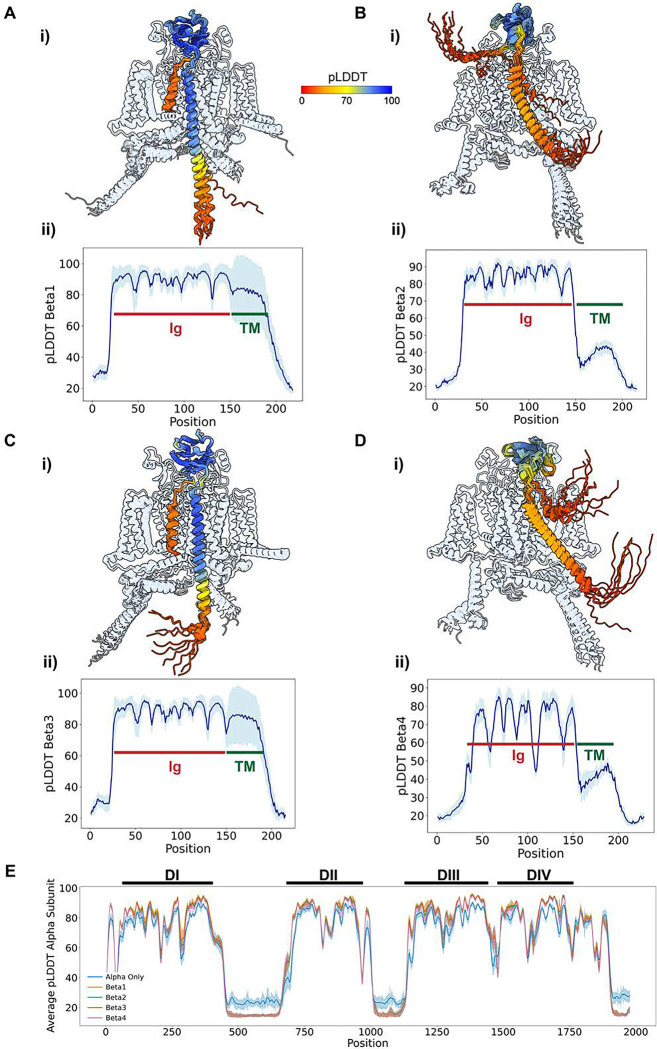
Distribution of pLDDT values across auxiliary β-subunits in complex with hNa_V_1.7. (A-D, i) Lateral view of top 10 models of hNa_V_1.7 in complex with the four auxiliary β-subunits; the α-subunit ribbon is showing with transparency and the auxiliary β-subunit ribbons are colored by pLDDT values. (A-D, ii) Average pLDDT values per position across the four auxiliary β-subunits in all generated models; shaded areas indicate the standard deviation. (E) Average pLDDT values per position across the α-subunit in all generated models with the α-subunit alone, or in complex with the four auxiliary β-subunits; shaded areas indicate the standard deviation.

When analyzing the distribution of pLDDT values across the four β-subunits, we observe that β1 and β3 have a similar distribution (Fig. 8 A and C). The immunoglobulin domain has very high pLDDT values, ~90, while the transmembrane domain maintains fairly high accuracy but exhibits more variability in pLDDT values. We also observe that the N-terminus of the protein, the signal peptide, is inserted into the membrane with low confidence, but we know this part is likely cleaved and doesn’t form part of the final structure. The end of the transmembrane domain in the intracellular region and not resolved in experimental structures, also has low confidence. For β2 and β4, we see a similar pattern between the two (Fig. 8 B and D). The immunoglobulin domain has higher pLDDT values, again ~80–90. For β4, we observe regions of the immunoglobulin domain with lower pLDDT values, suggesting that β4 immunoglobulin domain may exhibit more conformational flexibility. The difference we observe in the transmembrane domain—bearing in mind that, for β2 and β4, this domain is unresolved in experimental structures—is not only that it’s tilted with respect to the membrane axis but also that it shows significantly lower confidence, with pLDDT values dropping below 50, indicating high conformational flexibility. This likely explains why we don’t see these transmembrane domains in experimental structures.

The other question we addressed was whether the presence of the β-subunits affects the pLDDT distribution of the α-subunit (Fig. 8 E). For most of the structured regions of the α-subunit’s four domains, the presence of the β-subunits increases pLDDT compared to when the β-subunits are absent. This applies to the structured regions. However, in the unstructured intracellular loops, we see that when the α-subunit is alone, it has higher pLDDT, although these values are still quite low, below 30, indicating high uncertainty in the conformation of this entire region. Therefore, the presence of β-subunits appears to increase the pLDDT of the structured regions of the α-subunit, which may indicate conformational stabilization due to the β-subunits.

Our results show that β1 and β3 models exhibit similar characteristics, with high pLDDT values, particularly in the immunoglobulin domain. β2 and β4 also display similar patterns but have lower confidence in the transmembrane membrane region, indicating greater flexibility. Additionally, the presence of β auxiliary subunits seems to stabilize the structured regions of the α-subunit, increasing their pLDDT.

### Effect of recycles and ipTM values on Na_V_ α- β multimer complex modeling

The next aspect we wanted to investigate, knowing that high-accuracy models can be generated, was how these multimer models evolve over the course of six recycles and to analyze a confidence parameter beyond pLDDT—namely, ipTM. ipTM is a metric reported by AlphaFold Multimer that relates to the confidence in the interface between the different monomers that form the multimer complex and takes values from 0 (low confidence) to 1 (high confidence).

**Figure 9.**
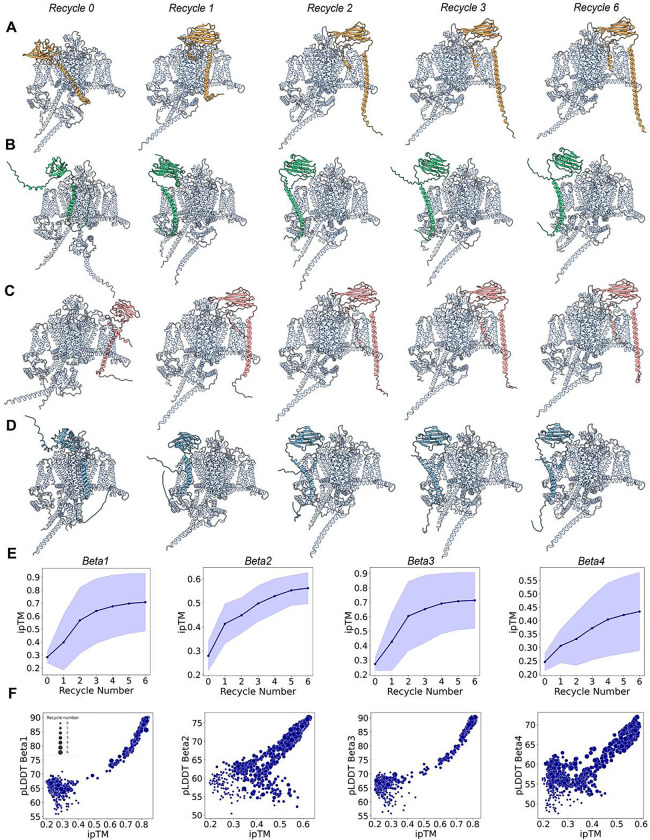
Analysis of the effect of recycles and ipTM values on Na_V_ α- β multimer complex modeling. (A-D) Top model of hNa_V_1.7 in complex with each of the four auxiliary β-subunits across recycles. (E) Evolution across recycles of the average iPTM values of all generated models for each of the four β-subunit cases; shaded areas indicate the standard deviation. (F) Relationship between model iPTM and β-subunit pLDDT values.

To conduct this analysis, we examined the top model for each of the four β-subunits in complex with hNa_V_1.7, observing how the structures evolved from recycle 0 to recycle 6 (Fig. 9 A–D). At recycle 0, the β-subunit is positioned in an improbable location with numerous steric clashes with the α-subunit. As the number of recycles progresses to recycles 1 and 2, the β-subunit gradually relocates to the appropriate region, either adjacent to VSDI or VSDIII, depending on the specific β-subunit. From Recycle 3 onwards, minimal structural changes occur, with no significant alterations observed up to Recycle 6 (Fig. 9 A–D; Video S4).

We next analyzed the ipTM values and their evolution over the number of recycles. Consistent with our visual observations, ipTM values increase significantly from Recycle 0 to 1, 2, and 3, after which they stabilize across all four β-subunits (Fig. 9 E). We also observed distinct differences between the four subunits. β1 and β3 achieve higher ipTM values, reaching up to 0.8 in some instances, whereas β2 and β4 have lower ipTM values ~0.5. Particularly, β4, which exhibited lower confidence and greater flexibility, also displayed the lowest ipTM values.

Finally, we investigated the relationship between ipTM values and the pLDDT of the β-subunit alone. As expected, a positive correlation was observed, with models that had higher ipTM values also demonstrating higher pLDDT values for the β-subunit (Fig. 9 F). For β1 and β2, there was a clearer trend towards higher ipTM and pLDDT values, while β2 and β3 showed more models within the intermediate ipTM range. In conclusion, the overall correlation pattern between these two variables—pLDDT and ipTM—was similar between β1 and β3, and likewise similar between β2 and β4, suggesting a pairwise similarity between β1 and β3, and between β2 and β4.

### Auxiliary β-subunits profoundly influence the conformational landscape of Na_V_ α-subunit, especially for VSDIV

**Figure 10.**
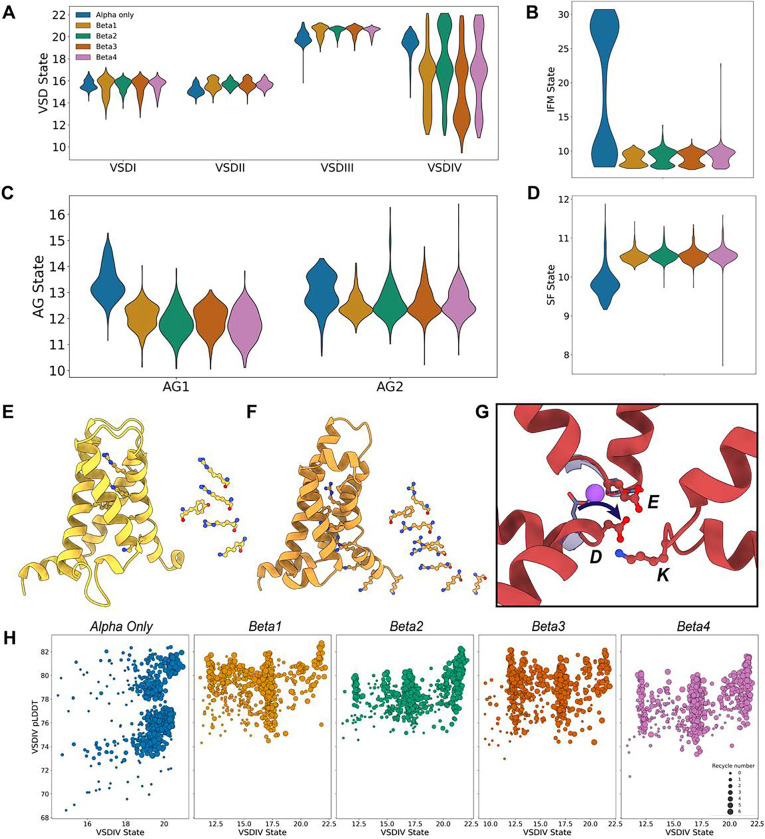
Effect of the presence of auxiliary β-subunits in α-subunit conformational distribution. (A-D) Comparison of the distribution of states of the VSDs (A), IFM motif (B), AG (C), and SF (D) in the presence of the four different β-subunits and when the α-subunit is modeled alone. (E) Model of hNa_V_1.7- β-1 that shows a VSDI in a more deactivated state than what was observed with the α-subunit alone. (F) Model of hNa_V_1.7- β-3 that shows the VSDIV in a state representing one additional “click” of translocation of gating charges across the hydrophobic constriction site from what was observed for the α-subunit alone. (G) Outlier model of hNa_V_1.7- β-4 that shows a contracted SF where the aspartate interacts with the lysine from the DEKA motif. (H) Relationship between VSDIV state distributions for each modeling case (4 β-subunits plus α-subunit alone) and the VSDIV pLDDT; dot sizes represent recycle number.

After analyzing the results presented so far, we sought to determine if the presence of the different β-subunits affects the distribution of conformations in the different regions of the α-subunit. We first examined the impact of the β-subunits on the distribution of states of the VSDs (Fig. 10 A). Overall, we found that the effect on VSDII and VSDIII was minimal, with very small differences in GC1-S4 – HC-S2 distance distributions. However, larger effects were observed in VSDI, where the presence of auxiliary subunits, particularly β1 and β3, led to more deactivated states being sampled. The most pronounced effect was observed in VSDIV, where the presence of β-subunits significantly altered the state distribution, with a much greater frequency of deactivated states with lower GC1-S4 – HC-S2 distances.

We then visually inspected models that might represent novel states introduced by the presence of the β-subunits in the modeling. In the model with VSDI in the most deactivated state, which corresponds to a model with β1, we observed a conformation similar to the most deactivated VSDI state seen with the α-subunit alone: only two gating charges were positioned above the hydrophobic constriction site (Fig. 10 E). However, compared to the most deactivated VSDI α-only model (Fig. 2 A), the third gating charge was positioned significantly lower relative to the hydrophobic constriction site in the model with β1. Interestingly, for the model with β3 where VSDIV was in its most deactivated state, we noted that this state was more deactivated than what was observed in the α-only model. In the α-only model, the most deactivated state reached had two gating charges above the hydrophobic constriction site, whereas in the β3 model, only one gating charge was positioned above the hydrophobic constriction site, which results in an additional “click” down to what we have previously observed (Video S5). This represents a state that has not been observed experimentally.

We observed a significant effect of the β-subunits on the IFM motif conformations distribution (Fig. 10 B). With the α-subunit alone, a bimodal distribution of the IFM motif being either bound or unbound was present. However, in the presence of the β-subunits, the IFM motif was more frequently modeled bound to the pore domain, suggesting that the presence of β-subunits biases the models towards an IFM-bound state.

Analysis of the AG (Fig. 10 C) revealed that the presence of β-subunits generally reduced the area of the activation gate, although certain outliers with larger areas appeared. For the SF (Fig. 10 D), we observed significant changes in the SF-D–K distance distribution, with the average distances being more dilated (~10.5 Å). Interestingly, for β4, we identified outlier models with a highly contracted selectivity filter, with distance coordinate values below 8 Å. Visual analysis of this outlier (Fig. 10 G; Video S6) suggests that the contraction is due to an interaction between the aspartic acid and lysine residues of the DEKA motif, disrupting sodium coordination. This interaction may represent another possible slow inactivated state.

Returning to VSDIV, we observed that the presence of β-subunits led to models spanning a broad range of states, from very deactivated to fully activated. This provided an opportunity to examine the relationship between pLDDT values and a broad range of VSDIV states (Fig. 10 H). The results revealed that the presence of β-subunits led to a complete redistribution of states in VSDIV compared to the α-only models. Notably, even the most deactivated states of VSDIV, when β-subunits were present, maintained high pLDDT values ~80, indicating that these deactivated models were generated with high confidence.

Our results show that the presence of β-subunits during AlphaFold modeling profoundly affects the conformational landscape of the α-subunit, resulting also in novel states not observed when modeling the α-subunit alone. Notably, we observed a similar reshaping of state distributions for hNa_V_1.1 (Fig. S1). This effect is particularly pronounced in VSDIV, where β-subunits lead to more deactivated states that maintain high confidence.

### Modeling Na_V_-CaM interactions resulted in sampling of both Ca^2+^-bound and Ca^2+^-free conformations

In the final part of this study, we explored the interaction between Na_V_ channel α-subunit and CaM, a Ca^2+^ sensor protein known to modulate Na_V_ channel activity. Particularly, CaM modulates channel inactivation by interacting with the α-subunit C-T region, specifically with an alpha-helical segment known as the IQ motif (Wu and Hong, 2021). Unlike the auxiliary β-subunits, CaM is a protein with greater conformational diversity, depending on the Ca^2+^ concentration, which makes this analysis more complex. Moreover, there is no experimental structure of a full Na_V_ channel bound to CaM, although partial structures with CaM bound to the IQ segment in the C-T domain of Na_V_ channels are available (Gabelli et al., 2014; Wang et al., 2014; Hovey et al., 2017). This analysis is also particularly interesting because AlphaFold2, does not support non-protein atoms, like cations. Therefore, Ca^2+^ was absent from our modeling; in the future, with newer versions of AlphaFold that support cations, more rigorous analysis could be conducted. In this case, we focused on two test cases: hNa_V_1.2 and hNa_V_1.5, modeling both with CaM using the same setup as before generating 100 models with six recycles.

**Figure 11.**
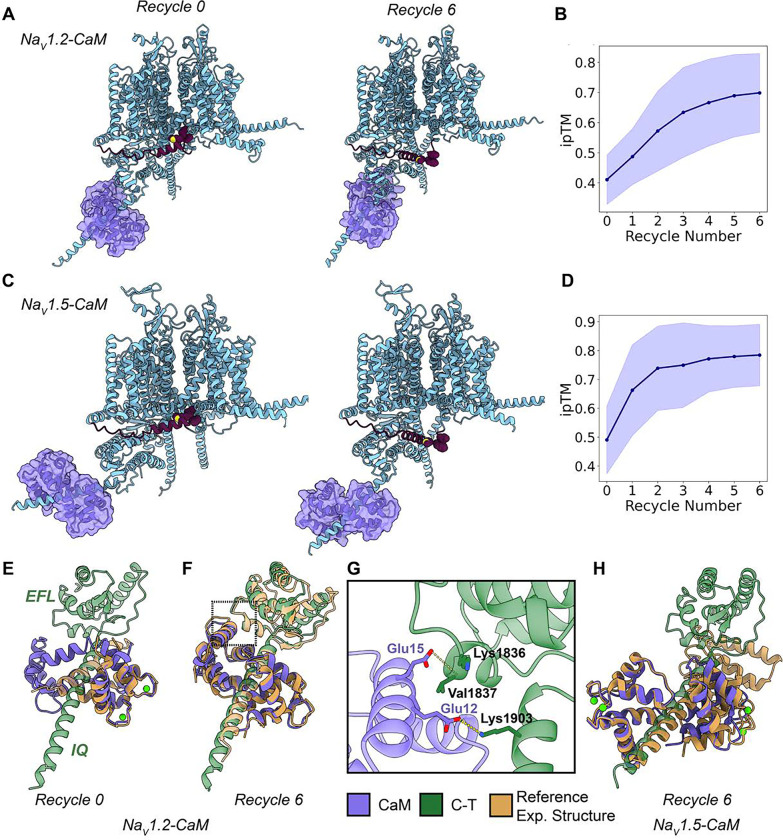
Analysis of top models generated with AlphaFold Multimer of CaM bound to hNa_V_1.2 and hNa_V_1.5. (A, C) Overview of CaM-α-subunit complex top models for hNa_V_1.2 (A) and hNa_V_1.5 (B) at recycle 0 and recycle 6. The IFM motif is highlighted in purple. Unstructured intracellular loops are hidden for clarity. (B, D) Evolution across recycles of the average iPTM values of all generated models of CaM bound to hNa_V_1.2 (B) and hNa_V_1.5 (D); shaded areas indicate the standard deviation. (E) Detailed view of top CaM-hNa_V_1.2 model (purple, green) at recycle 0 superimposed into the experimental structure (orange) of Ca^2+^-saturated-CaM bound to the IQ motif of Na_V_1.2 (PDB: 2M5E, (Hovey et al., 2017)). (F) Detailed view of top CaM-hNa_V_1.2 model (purple, green) at recycle 6 superimposed into the experimental structure (orange) of Ca^2+^-free-CaM bound to the IQ motif of Na_V_1.5 (PDB: 4OVN, (Gabelli et al., 2014)). (G) Interactions between CaM and the C-T of the α-subunit in the area highlighted with a dashed square in (F). (H) Detailed view of top CaM-hNa_V_1.5 model (purple, green) at recycle 6 superimposed into the experimental structure (orange) of Ca^2+^-saturated-CaM bound to the IQ motif of Na_V_1.5 (PDB: 4JQ0, (Wang et al., 2014)).

The first analysis involved visually analyzing the evolution of the top-ranked models over the six recycles for each case. The behavior of the IFM motif and CaM conformation revealed interesting differences between hNa_V_1.2 and hNa_V_1.5 (Fig. 11 A and C). Interestingly, for both channels, the IFM motif initially appears bound to the pore domain at recycle 0 but becomes unbound as recycles progress. Additionally, when analyzing how ipTM values evolve over the number of recycles, we observe that for hNa_V_1.2, this increase is more gradual and doesn’t stabilize until recycle 5 (Fig. 11 B). In contrast, for hNa_V_1.5, ipTM quickly stabilizes by recycle 2, reaching values ~0.8 (Fig. 11 D). For hNa_V_1.2, the ipTM values are slightly lower, staying ~0.7 on average.

Notably, CaM displayed conformational changes across recycles for hNa_V_1.2. We observed two distinct states between recycle 0 and recycle 6 (Fig. 11 E and F; Video S7). In recycle 0, the N-lobe of CaM adopts a conformation similar to the Ca^2+^-bound experimental structure (PDB: 2M5E, Ca^2+^-saturated-CaM bound to the IQ motif of Na_V_1.2) (Hovey et al., 2017), while the C-lobe does not. By recycle 6, CaM exhibits a conformation corresponding to the Ca^2+^-free experimental structure (PDB: 4OVN, Ca^2+^-free-CaM bound to the IQ motif of Na_V_1.5) (Gabelli et al., 2014), where the N-lobe interacts with the EFL domain of the C-T region of hNa_V_1.2 (Fig. 11 G). For hNa_V_1.5, CaM remains in the same conformation across recycles in the top model. Compared with the reference experimental structure (PDB: 4JQ0, Ca^2+^-saturated-CaM bound to the IQ motif of Na_V_1.5) (Wang et al., 2014), this CaM state resembles the fully Ca^2+^-bound conformation of CaM bound to the IQ motif (Fig. 11 H).

**Figure 12.**
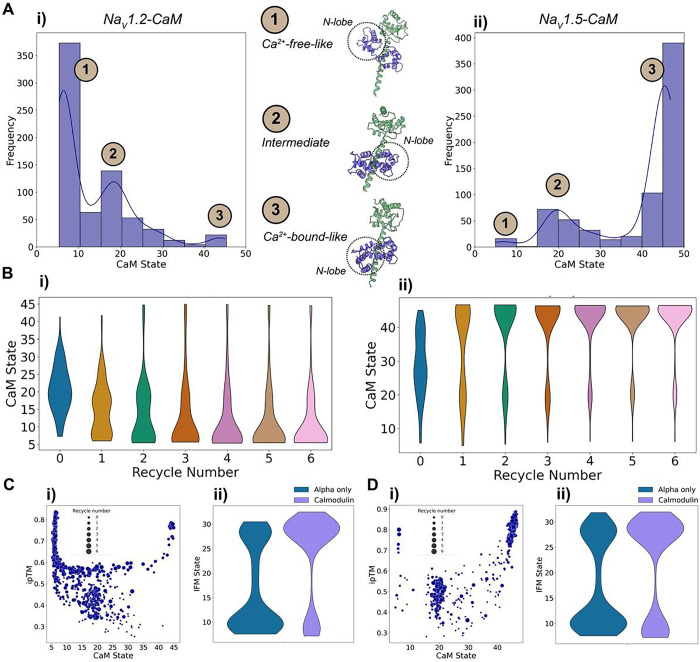
Analysis of state distributions of CaM and its effect on the α-subunit. (A) CaM state distributions defined by the CaM-Glu15 – CT distance for hNa_V_1.2 (i) and hNa_V_1.5 (ii); representative models of the three identified states are shown. (B) Evolution of CaM state distributions across recycles for hNa_V_1.2 (i) and hNa_V_1.5 (ii). (C,D) Relationship between CaM state and model iPTM (i) and IFM state distribution in the presence or absence of CaM (ii) for hNa_V_1.2 (C) and hNa_V_1.5 (D).

To further understand these differences, we defined a coordinate distance between Glu15 of CaM and a specific residue (Val1837 in hNa_V_1.2 and the equivalent Ile1833 in hNa_V_1.5) in the EFL domain of the α-subunit C-T domain (Fig. 11 G). This distance (CaM-Glu15 – CT) helps determine the CaM state: it is shorter in the Ca^2+^-free-like state when the N-lobe interacts with the C-terminal, and larger when CaM is in the Ca^2+^-bound-like state, where the N-lobe no longer interacts. Using this metric, we observed the distribution of CaM states for hNa_V_1.2 and hNa_V_1.5 (Fig. 12 A). Three distinct states emerged: (1) a Ca^2+^-free-like state with low CaM-Glu15 – CT distances, where the N-lobe interacts with the C-terminal; (2) an intermediate state, with one lobe Ca^2+^-bound-like and the other not; and (3) a fully Ca^2+^-bound-like state in both lobes. Interestingly, while hNa_V_1.2 and hNa_V_1.5 channels sampled all three states, there was a notable difference between the two channels. For hNa_V_1.2, the Ca^2+^-free-like conformation was predominant, whereas for hNa_V_1.5, the Ca^2+^-bound-like conformation was more frequently sampled. We also analyzed how these CaM states varied with the recycle number (Fig. 12 B). Consistent with previous observations, recycle 0 showed the greatest conformational diversity. As the number of recycles increased, CaM converged to a predominant state—the Ca^2+^-free state for hNa_V_1.2, and the Ca^2+^-bound state for hNa_V_1.5.

We also assessed the relationship between CaM states and ipTM values (Fig. 12 C and D, panel i). Extreme states (either Ca^2+^-free or Ca^2+^-bound) showed higher ipTM values. For hNa_V_1.2, the Ca^2+^-free state reached ipTM values above 0.8, while the Ca^2+^-bound state was slightly lower. Conversely, hNa_V_1.5’s Ca^2+^-bound state achieved ipTM values up to 0.9. These differences in maximum ipTM values may partly explain the preferential sampling of specific states for each Na_V_ channel. Lastly, we examined how the presence of CaM influences the distribution of IFM motif states (Fig. 12 C and D, panel ii). For both hNa_V_1.2 and hNa_V_1.5, the presence of CaM increased the sampling frequency of the unbound IFM motif state, as we expected from the visual analysis of the models. Notably, the presence of CaM also had an effect on the state distributions of other channel regions (Fig. S2).

Remarkably, AlphaFold2 predicts CaM’s interaction with Na_V_ channels in the absence of full experimental reference structures of Na_V_ channel – CaM complexes and the absence of Ca^2+^ ions in the modeling. Our models of Na_V_ channel – CaM complexes agree well with known structures of CaM in different states, but for a more definitive understanding, future modeling efforts incorporating Ca^2+^ ions will be essential.

## Discussion

With the emergence of deep learning methods, the field of structural biology has undergone a profound transformation (Baek and Baker, 2022). This evolution is largely driven by increased computational capabilities, particularly involving the development of advanced neural network algorithms, the massive accumulation of structural data that enables effective training of these models, and GPU-based computation. With the release of AlphaFold2 in 2020 (Jumper et al., 2021), the protein structure prediction accuracy significantly improved, although modeling protein folding remains challenging (Chen et al., 2023). Therefore, the next frontier in structural biology lies in predicting sets of protein structures that represent ensembles of conformations or states (Lane, 2023). This is because proteins are dynamic entities that undergo conformational changes, with varying degrees of complexity depending on their function. This problem of capturing different protein conformations inspired the addition of new section for predicting protein conformations in the Critical Assessment of Techniques for Protein Structure Prediction (CASP) in 2022 (Kryshtafovych et al., 2023). If protein sequence determines structure and structure represents an ensemble of conformations or states, then understanding the conformational ensemble, structurally and energetically, represents the last but most complex step to fully comprehend the structural changes a complex protein like an ion channel or a transporter undergoes during its functional cycle. This structural modeling challenge is directly relevant to Na_V_ channels, which are large proteins which open, inactivate, or close in response to electrical changes within cell membranes, which adds another layer of complexity.

We know that the energetic distributions of protein states are further influenced by interactions with additional partners, which may include other proteins, lipids, small molecules, cations, posttranslational modifications and more. This adds another dimension to the challenge, as understanding how these interactions influence the equilibrium of the different protein states is required for fully defining the protein’s function and regulation in its physiological context. Additionally, establishing how these interactions reshape the conformational landscape of a protein is particularly important for drug design. The most effective approach for achieving a targeted effect in drug development often involves focusing on a specific conformational state and determining how drug binding influences the protein’s function by altering its state distribution.

Several approaches are currently being explored to apply deep learning methods to solve the problem of predicting conformational distributions. First, some methods use deep learning to extract multiple conformations from experimental data (Ed et al., 2021; Punjani and Fleet, 2023; Wankowicz et al., 2024). Second, other deep learning methods aim to predict these ensembles of conformations using algorithms predominantly derived from AlphaFold, where modifications to the MSA construction are made to promote more diverse sampling (Stein and Mchaourab, 2022; Wayment-Steele et al., 2023; Sala et al., 2023; Monteiro da Silva et al., 2024).

In this study, we explored the capabilities of AlphaFold2 to model conformational diversity in different structural regions of Na_V_ channels by employing a subsampled MSA approach (Monteiro da Silva et al., 2024). Structural data has already provided a diverse view of Na_V_ channels conformational dynamics, and we aimed to assess how well the conformations generated by AlphaFold2 capture the diversity of states observed in experimental structures. Additionally, we used AlphaFold Multimer (Evans et al., 2022) to evaluate the ability of these methods to accurately model the interactions between the α-subunit of Na_V_ channels and protein partners, such as auxiliary Na_V_ β-subunits and CaM. We also examined how these interactions influence the conformational landscape of the α-subunit.

We first focused on modeling different states of regions of the α -subunit, specifically on seven specific areas: the four voltage-sensing domains (VSDs), the selectivity filter (SF), the activation gate (AG), and the IFM motif (Fig. 1). These regions are structurally well-characterized, and our goal was to determine whether AlphaFold2 and subsampled MSA approach could generate distinct conformations for these regions, comparing the results to available experimental data.

Overall, we found that AlphaFold2 generates diverse conformational states that agree with existing structural and mechanistic knowledge of Na_V_ channels. Specifically, for the VSDs, we observed at least two distinct states for each domain, with the gating charges in the S4 segment translocating by at least one “click” relative to the hydrophobic constriction site (Fig. 2). This pattern holds for each domain, and for the VSDIV in particular, we observed a wider sampling of two “clicks”. We questioned why there was greater conformational diversity sampled for the VSDIV compared to the others. One possible explanation is that experimental structures of Na_V_ channels capturing this conformation exist, particularly those where the VSDIV is trapped in a deactivated state by α-scorpion toxins (Clairfeuille et al., 2019; Shen et al., 2019). It is likely that AlphaFold2 is sampling these states because some structures in this state were present in its training set. However, although AlphaFold2 sampled two distinct states for the VSDII (with either three or two gating charges above the hydrophobic constriction site), it did not sample the more deactivated states seen in experimental structures of both chimeric constructs and full human Na_V_1.7 (Xu et al., 2019; Wisedchaisri et al., 2020; Huang et al., 2022a) with only one gating charge above the hydrophobic constriction site. Even with the use of custom templates, where specific templates with the VSDII in a deactivated conformation were used, we did not observe a significant effect on AlphaFold’s modeling of this domain (Fig. 5 I–K). Therefore, the underlying factors driving AlphaFold2 to model certain states in each VSD remain unclear. Additionally, for VSDI and VSDIII, it is possible that more deactivated states were not sampled, even though such states have not been observed experimentally. Electrophysiology experiments suggest that at least 12 charges must move during Na_V_ channel activation (Hirschberg et al., 1995), implying that each VSD could potentially exhibit up to three distinct “clicks” of gating charge translocations. Despite these limitations, AlphaFold2 models could offer valuable insights into the molecular transitions involved in each VSD activation and deactivation, as well as provide a foundation for state-specific targeting in drug development. Another important aspect of the distributions we observed for the VSDs is that they are fairly consistent across the nine Na_V_ subtypes. Interestingly, we observed that hNa_X_ exhibited a distinct distribution of states in the VSDs, SF, AG, and IFM motif. Given that hNa_X_ is not a voltage-sensing channel, we expected a different distribution compared to the other nine Na_V_ subtypes, and this was indeed observed. However, it remains unclear how to further interpret the differences observed between hNa_V_ and hNa_X_ states sampled by AlphaFold2.

For the IFM motif, we observed both states described in experimental data: the IFM motif in bound and unbound states (Fig. 3 A and B). The distributions of the IFM motif states varied between different channels, but it remains unclear whether these variations could potentially be related to the different inactivation patterns observed among the nine channel subtypes. It is considered that slow inactivation of Na_V_ channels involves conformational changes in the SF region, though this is less understood in mammalian Na_V_ channels (Fig. 3 C–E). Recent structures revealed new insights into minor conformational shifts in the SF that could render the channel non-conductive (Chen et al., 2024). AlphaFold2 primarily sampled the SF conformations consistent with experimental structures in a conductive state. However, we observed sampling with lower-frequency conformations of the aspartic acid of the DEKA motif that are not captured experimentally, which hypothetically could represent slow-inactivated states. Incorporating sodium ions in future modeling efforts using advanced tools like AlphaFold3 (Abramson et al., 2024) and RoseTTAFold All-Atom (Krishna et al., 2024) could provide more physiologically relevant insights into SF conformational changes and their role in slow inactivation. For the AG (Fig. 4), we observed a range of open and closed states for all channels. We also observed outliers where the AG appeared significantly open, suggesting improbable or rare conformations that may be short-lived. These findings highlight AlphaFold2’s ability to generate a diversity of AG states, which is experimentally challenging to capture due to the IFM motif bound conformation stabilizing an inactivated state in most Na_V_ structures (Table S2). Open states have been experimentally resolved in Na_V_ structures where the IFM motif is replaced with three glutamines (QQQ mutant) (Jiang et al., 2021a). Thus, our models may provide insights into AG dynamics and serve to formulate structural hypotheses for future experimental validation.

The pLDDT is a metric reported by AlphaFold2 that indicates the confidence of the generated models at the residue backbone level. In some cases, it has been linked to conformational flexibility (Wilson et al., 2022), though some studies show there isn’t a clear correlation between pLDDT and experimental B-factors, that represent atomic mobility or uncertainty in experimental structures (Carugo, 2023). Nevertheless, it’s one of the primary approaches to evaluate models generated by AlphaFold2. We observed specific distributions of pLDDT for each of the state coordinates of these regions (Fig. 5 A–G). Understanding the relationship between pLDDT, state distributions, and the actual energetic landscape of the protein states is challenging, and it remains uncertain if this information can be directly obtained from AlphaFold2-generated data. However, one clear conclusion from our results is that the greatest conformational diversity occurs at recycle 0 (Fig. 5 I–L). As recycle number increases, the structures tend toward specific states with high pLDDT values. This raises the question of whether AlphaFold2 sampling of specific states with increased recycles occurs because these states are more commonly seen in the training set or if these states are more energetically stable. However, this is a complex issue since the states we often observe in experimental structures are typically those that are also the most energetically stable.

The α-subunit forms the core of the Na_V_ channels, but under physiological conditions, it is finely regulated by protein partners (Namadurai et al., 2015). The most relevant partners are the auxiliary β-subunits, although the α-subunit also responds to intracellular signals mediated by other proteins, such as CaM, a Ca^2+^sensor (Wu and Hong, 2021). In our modeling of hNa_V_1.7 and hNa_V_1.1 channels with the four β-subunits, we successfully generated high-accuracy models that agreed well with experimental structures (Fig. 7). This demonstrates that AlphaFold Multimer is capable of accurately sampling the complexes between the α-subunit and β-subunits. Although there is a disulfide bond attaching β-2 and β-4 to the α-subunit, AlphaFold2 does not model covalent bonds, and the cysteines involved in these bonds are positioned close enough in the models for potential formation of a disulfide bond. The distribution of pLDDT values (Fig. 8) is particularly noteworthy for the transmembrane domains of β-2 and β-4, which show very low pLDDT values (~30–40). While the transmembrane domains of β-2 and β-4 have not been resolved experimentally, AlphaFold Multimer models predict that they span the membrane, although with a slight tilt relative to the membrane’s normal axis. This could be attributed to increased flexibility of these regions, although other factors, such as other protein partners and membrane lipid composition, might also contribute. Our results also demonstrate that the presence of the β-subunits reshapes the modeled conformational landscape of the α-subunit (Fig. 10). In particular, we were able to generate models of VSDIV with more deactivated states than what has been observed experimentally and when modeling the α-subunit alone. These data reveal possible redistribution of the Na_V_ α-subunit conformational landscape by the presence of the auxiliary β-subunits that may modulate channel activity. We see a similar pattern in the case of Na_V_ α-subunit complexes with CaM, where the IFM motif state shows a clear redistribution in the presence of CaM.

For CaM, our models agree well with reference experimental structures of Na_V_ channel regions in complex with CaM (Fig. 11). However, currently there are no structures available of a full Na_V_ channel in a complex with CaM. Additionally, modeling CaM presents a challenge due to its Ca^2+^-regulated conformational diversity. We observed conformational changes across the recycles, with a tendency toward different states in the two cases tested (Fig. 12). Specifically, for hNa_V_1.2, the Ca^2+^-free state was most frequently sampled, whereas for hNa_V_1.5, the Ca^2+^-bound state was more common. However, it is challenging to make conclusions about how these differences could relate to the subtype-specific interaction effects of CaM without a more in-depth analysis. Despite these limitations, the AlphaFold Multimer models of Na_V_ – CaM complexes might be useful for structural hypothesis formulation and guiding experimental studies aimed at deciphering the molecular mechanisms of Na_V_ channel CaM-dependent modulation. The next step would involve incorporating Ca^2+^ into the modeling, which is possible with AlphaFold3 (Abramson et al., 2024) and RoseTTAFold All-Atom (Krishna et al., 2024).

Our study has demonstrated that AlphaFold2 can generate different conformations of Na_V_ channels across various regions and accurately model interactions with protein partners of the α-subunit. We also showed that the presence of these partners impacts both the accuracy and the state distribution of the α-subunit. However, we do not claim that all of the Na_V_ states observed necessarily reflect the physiological distribution of states. Instead, we demonstrate and describe the capability to model different conformations, providing a starting point for formulating structural hypotheses that can be experimentally tested and further investigated to determine if the molecular mechanisms suggested by the models have actual physiological significance. An important remark is that while obtaining structures representing conformational ensembles is important, the ultimate goal would be to translate these distributions into actual energetic parameter predictions, such as channel gating kinetics, which would allow us to derive deeper functional insights. Additionally, modeling different channel conformations has significant implications for drug discovery, as structures with specific conformations can serve as inputs for designing *de novo* proteins or small molecule drugs that target particular states of voltage-gated ion channels. We are currently applying novel deep-learning methods for the design of *de novo* proteins that specifically target different regions and conformational states of voltage-gated ion channels (Mateos et al., 2024).

In conclusion, while our understanding of the full capabilities of these deep learning models for modeling protein conformational states is still developing, it is clear that they hold significant potential. Extensive use and thorough study are required to explore their capabilities, continuously validating and comparing them with experimental data. Many of the conformational transitions observed in this study remain inaccessible to traditional computational physics-based methods, such as molecular dynamics, due to the timescales involved. Deep learning methods hold promise for addressing these challenges. This is a rapidly advancing field, with new methods being developed to model diverse conformations and increase the sampling diversity of protein structure prediction methods. Moreover, newer deep learning methods are actively emerging, offering improved accuracy and the ability to include heteroatoms, which opens up possibilities for studying proteins in broader contexts (Abramson et al., 2024; Krishna et al., 2024). This holds particular promise for Na_V_ channels to advance our understanding of their molecular mechanisms and potential to design potent and selective channel modulators with therapeutic applicability (Mateos et al., 2024).

## Supplementary Material

Supplement 1

## Data Availability

All the models generated in this study for all test cases are openly available in the Dryad repository: https://doi.org/10.5061/dryad.rn8pk0pn3. (Active link for Reviewers: http://datadryad.org/stash/share/XjQoAjQ7urq1GsHCu1falyK7Rwe_lqobGJEsQPTZhSI)

## References

[R1] Abramson J., Adler J., Dunger J., Evans R., Green T., Pritzel A., Ronneberger O., Willmore L., Ballard A.J., Bambrick J., Bodenstein S.W., Evans D.A., Hung C.-C., O’Neill M., Reiman D., Tunyasuvunakool K., Wu Z., Žemgulytė A., Arvaniti E., Beattie C., Bertolli O., Bridgland A., Cherepanov A., Congreve M., Cowen-Rivers A.I., Cowie A., Figurnov M., Fuchs F.B., Gladman H., Jain R., Khan Y.A., Low C.M.R., Perlin K., Potapenko A., Savy P., Singh S., Stecula A., Thillaisundaram A., Tong C., Yakneen S., Zhong E.D., Zielinski M., Žídek A., Bapst V., Kohli P., Jaderberg M., Hassabis D., and Jumper J.M.. 2024. Accurate structure prediction of biomolecular interactions with AlphaFold 3. Nature. 1–3. doi:10.1038/s41586-024-07487-w. PMC11168924 38718835

[R2] del Alamo D., Sala D., Mchaourab H.S., and Meiler J.. 2022. Sampling alternative conformational states of transporters and receptors with AlphaFold2. eLife. 11:e75751. doi:10.7554/eLife.75751. 35238773 PMC9023059

[R3] Alsaloum M., Higerd G.P., Effraim P.R., and Waxman S.G.. 2020. Status of peripheral sodium channel blockers for non-addictive pain treatment. Nat Rev Neurol. 16:689–705. doi:10.1038/s41582-020-00415-2. 33110213

[R4] Baek M., and Baker D.. 2022. Deep learning and protein structure modeling. Nat Methods. 19:13–14. doi:10.1038/s41592-021-01360-8. 35017724

[R5] Baek M., DiMaio F., Anishchenko I., Dauparas J., Ovchinnikov S., Lee G.R., Wang J., Cong Q., Kinch L.N., Schaeffer R.D., Millán C., Park H., Adams C., Glassman C.R., DeGiovanni A., Pereira J.H., Rodrigues A.V., van Dijk A.A., Ebrecht A.C., Opperman D.J., Sagmeister T., Buhlheller C., Pavkov-Keller T., Rathinaswamy M.K., Dalwadi U., Yip C.K., Burke J.E., Garcia K.C., Grishin N.V., Adams P.D., Read R.J., and Baker D.. 2021. Accurate prediction of protein structures and interactions using a three-track neural network. Science. 373:871–876. doi:10.1126/science.abj8754. 34282049 PMC7612213

[R6] Bagal S.K., Brown A.D., Cox P.J., Omoto K., Owen R.M., Pryde D.C., Sidders B., Skerratt S.E., Stevens E.B., Storer R.I., and Swain N.A.. 2013. Ion channels as therapeutic targets: a drug discovery perspective. J Med Chem. 56:593–624. doi:10.1021/jm3011433. 23121096

[R7] Bennett D.L.H., and Woods C.G.. 2014. Painful and painless channelopathies. Lancet Neurol. 13:587–599. doi:10.1016/S1474-4422(14)70024-9. 24813307

[R8] Bosmans F., and Tytgat J.. 2007. Voltage-gated sodium channel modulation by scorpion α-toxins. Toxicon. 49:142–158. doi:10.1016/j.toxicon.2006.09.023. 17087986 PMC1808227

[R9] Capes D.L., Goldschen-Ohm M.P., Arcisio-Miranda M., Bezanilla F., and Chanda B.. 2013. Domain IV voltage-sensor movement is both sufficient and rate limiting for fast inactivation in sodium channels. J Gen Physiol. 142:101–112. doi:10.1085/jgp.201310998. 23858005 PMC3727307

[R10] Carugo O. 2023. pLDDT Values in AlphaFold2 Protein Models Are Unrelated to Globular Protein Local Flexibility. Crystals. 13:1560. doi:10.3390/cryst13111560.

[R11] Catterall W.A. 2023. Voltage gated sodium and calcium channels: Discovery, structure, function, and Pharmacology. Channels. 17:2281714. doi:10.1080/19336950.2023.2281714. 37983307 PMC10761118

[R12] Catterall W.A., Lenaeus M.J., and Gamal El-Din T.M.. 2020a. Structure and Pharmacology of Voltage-Gated Sodium and Calcium Channels. Annu Rev Pharmacol Toxicol. 60:133–154. doi:10.1146/annurev-pharmtox-010818-021757. 31537174

[R13] Catterall W.A., Wisedchaisri G., and Zheng N.. 2020b. The conformational cycle of a prototypical voltage-gated sodium channel. Nat Chem Biol. 16:1314–1320. doi:10.1038/s41589-020-0644-4. 33199904 PMC7678813

[R14] Chanda B., and Bezanilla F.. 2002. Tracking voltage-dependent conformational changes in skeletal muscle sodium channel during activation. J Gen Physiol. 120:629–645. doi:10.1085/jgp.20028679. 12407076 PMC2229551

[R15] Chaudhury S., Lyskov S., and Gray J.J.. 2010. PyRosetta: a script-based interface for implementing molecular modeling algorithms using Rosetta. Bioinformatics. 26:689–691. doi:10.1093/bioinformatics/btq007. 20061306 PMC2828115

[R16] Chen H., Xia Z., Dong J., Huang B., Zhang J., Zhou F., Yan R., Shi Y., Gong J., Jiang J., Huang Z., and Jiang D.. 2024. Structural mechanism of voltage-gated sodium channel slow inactivation. Nat Commun. 15:3691. doi:10.1038/s41467-024-48125-3. 38693179 PMC11063143

[R17] Chen S.-J., Hassan M., Jernigan R.L., Jia K., Kihara D., Kloczkowski A., Kotelnikov S., Kozakov D., Liang J., Liwo A., Matysiak S., Meller J., Micheletti C., Mitchell J.C., Mondal S., Nussinov R., Okazaki K., Padhorny D., Skolnick J., Sosnick T.R., Stan G., Vakser I., Zou X., and Rose G.D.. 2023. Protein folds vs. protein folding: Differing questions, different challenges. Proceedings of the National Academy of Sciences. 120:e2214423119. doi:10.1073/pnas.2214423119. PMC9910419 36580595

[R18] Clairfeuille T., Cloake A., Infield D.T., Llongueras J.P., Arthur C.P., Li Z.R., Jian Y., Martin-Eauclaire M.-F., Bougis P.E., Ciferri C., Ahern C.A., Bosmans F., Hackos D.H., Rohou A., and Payandeh J.. 2019. Structural basis of α-scorpion toxin action on Nav channels. Science. 363. doi:10.1126/science.aav8573. 30733386

[R19] Ed Z., B. T, B. B, and Jh D.. 2021. CryoDRGN: reconstruction of heterogeneous cryo-EM structures using neural networks. Nature methods. 18. doi:10.1038/s41592-020-01049-4. PMC8183613 33542510

[R20] Evans R., O’Neill M., Pritzel A., Antropova N., Senior A., Green T., Žídek A., Bates R., Blackwell S., Yim J., Ronneberger O., Bodenstein S., Zielinski M., Bridgland A., Potapenko A., Cowie A., Tunyasuvunakool K., Jain R., Clancy E., Kohli P., Jumper J., and Hassabis D.. 2022. Protein complex prediction with AlphaFold-Multimer. bioRxiv. 2021.10.04.463034. doi:10.1101/2021.10.04.463034.

[R21] Fan X., Huang J., Jin X., and Yan N.. 2023. Cryo-EM structure of human voltage-gated sodium channel Nav1.6. Proceedings of the National Academy of Sciences. 120:e2220578120. doi:10.1073/pnas.2220578120. PMC9945969 36696443

[R22] Gabelli S.B., Boto A., Kuhns V.H., Bianchet M.A., Farinelli F., Aripirala S., Yoder J., Jakoncic J., Tomaselli G.F., and Amzel L.M.. 2014. Regulation of the NaV1.5 cytoplasmic domain by calmodulin. Nat Commun. 5:5126. doi:10.1038/ncomms6126. 25370050 PMC4223872

[R23] Goddard T.D., Huang C.C., Meng E.C., Pettersen E.F., Couch G.S., Morris J.H., and Ferrin T.E.. 2018. UCSF ChimeraX: Meeting modern challenges in visualization and analysis. Protein Sci. 27:14–25. doi:10.1002/pro.3235. 28710774 PMC5734306

[R24] Hirschberg B., Rovner A., Lieberman M., and Patlak J.. 1995. Transfer of twelve charges is needed to open skeletal muscle Na+ channels. Journal of General Physiology. 106:1053–1068. doi:10.1085/jgp.106.6.1053. 8786350 PMC2229305

[R25] Hovey L., Fowler C.A., Mahling R., Lin Z., Miller M.S., Marx D.C., Yoder J.B., Kim E.H., Tefft K.M., Waite B.C., Feldkamp M.D., Yu L., and Shea M.A.. 2017. Calcium triggers reversal of calmodulin on nested anti-parallel sites in the IQ motif of the neuronal voltage-dependent sodium channel NaV1.2. Biophysical Chemistry. 224:1–19. doi:10.1016/j.bpc.2017.02.006. 28343066 PMC5503752

[R26] Huang G., Liu D., Wang W., Wu Q., Chen J., Pan X., Shen H., and Yan N.. 2022a. High-resolution structures of human Nav1.7 reveal gating modulation through α-π helical transition of S6IV. Cell Reports. 39:110735. doi:10.1016/j.celrep.2022.110735. 35476982

[R27] Huang X., Jin X., Huang G., Huang J., Wu T., Li Z., Chen J., Kong F., Pan X., and Yan N.. 2022b. Structural basis for high-voltage activation and subtype-specific inhibition of human Nav1.8. Proceedings of the National Academy of Sciences. 119:e2208211119. doi:10.1073/pnas.2208211119. PMC9335304 35858452

[R28] Jiang D., Banh R., Gamal El-Din T.M., Tonggu L., Lenaeus M.J., Pomès R., Zheng N., and Catterall W.A.. 2021a. Open-state structure and pore gating mechanism of the cardiac sodium channel. Cell. 184:5151–5162.e11. doi:10.1016/j.cell.2021.08.021. 34520724 PMC8673466

[R29] Jiang D., Tonggu L., Gamal El-Din T.M., Banh R., Pomès R., Zheng N., and Catterall W.A.. 2021b. Structural basis for voltage-sensor trapping of the cardiac sodium channel by a deathstalker scorpion toxin. Nature Communications. 12:128. doi:10.1038/s41467-020-20078-3. PMC7782738 33397917

[R30] Jones J., Correll D.J., Lechner S.M., Jazic I., Miao X., Shaw D., Simard C., Osteen J.D., Hare B., Beaton A., Bertoch T., Buvanendran A., Habib A.S., Pizzi L.J., Pollak R.A., Weiner S.G., Bozic C., Negulescu P., and White P.F.. 2023. Selective Inhibition of NaV1.8 with VX-548 for Acute Pain. New England Journal of Medicine. 389:393–405. doi:10.1056/NEJMoa2209870. 37530822

[R31] Jumper J., Evans R., Pritzel A., Green T., Figurnov M., Ronneberger O., Tunyasuvunakool K., Bates R., Žídek A., Potapenko A., Bridgland A., Meyer C., Kohl S.A.A., Ballard A.J., Cowie A., Romera-Paredes B., Nikolov S., Jain R., Adler J., Back T., Petersen S., Reiman D., Clancy E., Zielinski M., Steinegger M., Pacholska M., Berghammer T., Bodenstein S., Silver D., Vinyals O., Senior A.W., Kavukcuoglu K., Kohli P., and Hassabis D.. 2021. Highly accurate protein structure prediction with AlphaFold. Nature. 596:583–589. doi:10.1038/s41586-021-03819-2. 34265844 PMC8371605

[R32] KrishnaR., WangJ., AhernW., SturmfelsP., VenkateshP., KalvetI., LeeG.R., Morey-BurrowsF.S., AnishchenkoI., HumphreysI.R., McHughR., VafeadosD., LiX., SutherlandG.A., HitchcockA., HunterC.N., KangA., BrackenbroughE., BeraA.K., BaekM., DiMaioF., and BakerD.. 2024. Generalized biomolecular modeling and design with RoseTTAFold All-Atom. Science. 384:eadl2528. doi:10.1126/science.adl2528.38452047

[R33] Kryshtafovych A., Montelione G.T., Rigden D.J., Mesdaghi S., Karaca E., and Moult J.. 2023. Breaking the conformational ensemble barrier: Ensemble structure modeling challenges in CASP15. Proteins. 91:1903–1911. doi:10.1002/prot.26584. 37872703 PMC10840738

[R34] Lane T.J. 2023. Protein structure prediction has reached the single-structure frontier. Nat Methods. 20:170–173. doi:10.1038/s41592-022-01760-4. 36639584 PMC9839224

[R35] Lee C.-H., and MacKinnon R.. 2019. Voltage Sensor Movements during Hyperpolarization in the HCN Channel. Cell. 179:1582–1589.e7. doi:10.1016/j.cell.2019.11.006. 31787376 PMC6911011

[R36] Li X., Xu F., Xu H., Zhang S., Gao Y., Zhang H., Dong Y., Zheng Y., Yang B., Sun J., Zhang X.C., Zhao Y., and Jiang D.. 2022. Structural basis for modulation of human NaV1.3 by clinical drug and selective antagonist. Nat Commun. 13:1286. doi:10.1038/s41467-022-28808-5. 35277491 PMC8917200

[R37] Li Z., Jin X., Wu T., Huang G., Wu K., Lei J., Pan X., and Yan N.. 2021a. Structural Basis for Pore Blockade of the Human Cardiac Sodium Channel Nav1.5 by the Antiarrhythmic Drug Quinidine**. Angewandte Chemie International Edition. 60:11474–11480. doi:10.1002/anie.202102196. 33684260

[R38] Li Z., Jin X., Wu T., Zhao X., Wang W., Lei J., Pan X., and Yan N.. 2021b. Structure of human Nav1.5 reveals the fast inactivation-related segments as a mutational hotspot for the long QT syndrome. PNAS. 118. doi:10.1073/pnas.2100069118. PMC7980460 33712541

[R39] Li Z., Wu Q., and Yan N.. 2024. A structural atlas of druggable sites on Nav channels. Channels. 18:2287832. doi:10.1080/19336950.2023.2287832. 38033122 PMC10732651

[R40] Lin Z., Akin H., Rao R., Hie B., Zhu Z., Lu W., Smetanin N., Verkuil R., Kabeli O., Shmueli Y., dos Santos Costa A., Fazel-Zarandi M., Sercu T., Candido S., and Rives A.. 2023. Evolutionary-scale prediction of atomic-level protein structure with a language model. Science. 379:1123–1130. doi:10.1126/science.ade2574. 36927031

[R41] Mandala V.S., and MacKinnon R.. 2022. Voltage-sensor movements in the Eag Kv channel under an applied electric field. Proc Natl Acad Sci U S A. 119:e2214151119. doi:10.1073/pnas.2214151119. 36331999 PMC9674223

[R42] Mandala V.S., and MacKinnon R.. 2023. The membrane electric field regulates the PIP2-binding site to gate the KCNQ1 channel. Proc Natl Acad Sci U S A. 120:e2301985120. doi:10.1073/pnas.2301985120. 37192161 PMC10214144

[R43] Mateos D.L., Harris B.J., González A.H., Narang K., and Yarov-Yarovoy V.. 2024. Harnessing Deep Learning Methods for Voltage-Gated Ion Channel Drug Discovery. Physiology. doi:10.1152/physiol.00029.2024. PMC11918310 39189871

[R44] Meisler M.H., Hill S.F., and Yu W.. 2021. Sodium channelopathies in neurodevelopmental disorders. Nat Rev Neurosci. 22:152–166. doi:10.1038/s41583-020-00418-4. 33531663 PMC8710247

[R45] Mirdita M., Schütze K., Moriwaki Y., Heo L., Ovchinnikov S., and Steinegger M.. 2022. ColabFold: making protein folding accessible to all. Nat Methods. 19:679–682. doi:10.1038/s41592-022-01488-1. 35637307 PMC9184281

[R46] Monteiro da Silva G., Cui J.Y., Dalgarno D.C., Lisi G.P., and Rubenstein B.M.. 2024. High-throughput prediction of protein conformational distributions with subsampled AlphaFold2. Nat Commun. 15:2464. doi:10.1038/s41467-024-46715-9. 38538622 PMC10973385

[R47] Muroi Y., Arcisio-Miranda M., Chowdhury S., and Chanda B.. 2010. Molecular determinants of coupling between the domain III voltage sensor and pore of a sodium channel. Nat Struct Mol Biol. 17:230–237. doi:10.1038/nsmb.1749. 20118934 PMC2879147

[R48] Namadurai S., Yereddi N.R., Cusdin F.S., Huang C.L.H., Chirgadze D.Y., and Jackson A.P.. 2015. A new look at sodium channel β subunits. Open Biol. 5:140192. doi:10.1098/rsob.140192. 25567098 PMC4313373

[R49] Nguyen P.T., Harris B.J., Mateos D.L., González A.H., Murray A.M., and Yarov-Yarovoy V.. 2024. Structural modeling of ion channels using AlphaFold2, RoseTTAFold2, and ESMFold. Channels. 18:2325032. doi:10.1080/19336950.2024.2325032. 38445990 PMC10936637

[R50] Nguyen P.T., and Yarov-Yarovoy V.. 2022. Towards Structure-Guided Development of Pain Therapeutics Targeting Voltage-Gated Sodium Channels. Front Pharmacol. 13:842032. doi:10.3389/fphar.2022.842032. 35153801 PMC8830516

[R51] Noland C.L., Chua H.C., Kschonsak M., Heusser S.A., Braun N., Chang T., Tam C., Tang J., Arthur C.P., Ciferri C., Pless S.A., and Payandeh J.. 2022. Structure-guided unlocking of NaX reveals a non-selective tetrodotoxin-sensitive cation channel. Nat Commun. 13:1416. doi:10.1038/s41467-022-28984-4. 35301303 PMC8931054

[R52] Noreng S., Li T., and Payandeh J.. 2021. Structural Pharmacology of Voltage-Gated Sodium Channels. Journal of Molecular Biology. 433:166967. doi:10.1016/j.jmb.2021.166967. 33794261

[R53] Pakhrin S.C., Shrestha B., Adhikari B., and Kc D.B.. 2021. Deep Learning-Based Advances in Protein Structure Prediction. Int J Mol Sci. 22:5553. doi:10.3390/ijms22115553. 34074028 PMC8197379

[R54] Pan X., Li Z., Huang X., Huang G., Gao S., Shen H., Liu L., Lei J., and Yan N.. 2019. Molecular basis for pore blockade of human Na+ channel Nav1.2 by the μ-conotoxin KIIIA. Science. 363:1309–1313. doi:10.1126/science.aaw2999. 30765605

[R55] Pan X., Li Z., Jin X., Zhao Y., Huang G., Huang X., Shen Z., Cao Y., Dong M., Lei J., and Yan N.. 2021. Comparative structural analysis of human Nav1.1 and Nav1.5 reveals mutational hotspots for sodium channelopathies. PNAS. 118. doi:10.1073/pnas.2100066118. PMC7980448 33712547

[R56] Pan X., Li Z., Zhou Q., Shen H., Wu K., Huang X., Chen J., Zhang J., Zhu X., Lei J., Xiong W., Gong H., Xiao B., and Yan N.. 2018. Structure of the human voltage-gated sodium channel Nav1.4 in complex with β1. Science. 362. doi:10.1126/science.aau2486. 30190309

[R57] Pravda L., Sehnal D., Toušek D., Navrátilová V., Bazgier V., Berka K., Svobodová Vařeková R., Koča J., and Otyepka M.. 2018. MOLEonline: a web-based tool for analyzing channels, tunnels and pores (2018 update). Nucleic Acids Research. 46:W368–W373. doi:10.1093/nar/gky309. 29718451 PMC6030847

[R58] Punjani A., and Fleet D.J.. 2023. 3DFlex: determining structure and motion of flexible proteins from cryo-EM. Nat Methods. 20:860–870. doi:10.1038/s41592-023-01853-8. 37169929 PMC10250194

[R59] Remme C.A., and Wilde A.A.M.. 2014. Targeting sodium channels in cardiac arrhythmia. Curr Opin Pharmacol. 15:53–60. doi:10.1016/j.coph.2013.11.014. 24721654

[R60] Sala D., Engelberger F., Mchaourab H.S., and Meiler J.. 2023. Modeling conformational states of proteins with AlphaFold. Curr Opin Struct Biol. 81:102645. doi:10.1016/j.sbi.2023.102645. 37392556

[R61] Shen H., Li Z., Jiang Y., Pan X., Wu J., Cristofori-Armstrong B., Smith J.J., Chin Y.K.Y., Lei J., Zhou Q., King G.F., and Yan N.. 2018. Structural basis for the modulation of voltage-gated sodium channels by animal toxins. Science. 362. doi:10.1126/science.aau2596. 30049784

[R62] Shen H., Liu D., Wu K., Lei J., and Yan N.. 2019. Structures of human Nav1.7 channel in complex with auxiliary subunits and animal toxins. Science. 363:1303–1308. doi:10.1126/science.aaw2493. 30765606

[R63] Shen H., Zhou Q., Pan X., Li Z., Wu J., and Yan N.. 2017. Structure of a eukaryotic voltage-gated sodium channel at near-atomic resolution. Science. 355:eaal4326. doi:10.1126/science.aal4326. 28183995

[R64] Sokolov S., Kraus R.L., Scheuer T., and Catterall W.A.. 2008. Inhibition of sodium channel gating by trapping the domain II voltage sensor with protoxin II. Mol. Pharmacol. 73:1020–1028. doi:10.1124/mol.107.041046. 18156314

[R65] Stein R.A., and Mchaourab H.S.. 2022. SPEACH_AF: Sampling protein ensembles and conformational heterogeneity with Alphafold2. PLoS Comput Biol. 18:e1010483. doi:10.1371/journal.pcbi.1010483. 35994486 PMC9436118

[R66] Vilin Y.Y., and Ruben P.C.. 2001. Slow inactivation in voltage-gated sodium channels. Cell Biochem Biophys. 35:171–190. doi:10.1385/CBB:35:2:171. 11892790

[R67] Wang C., Chung B.C., Yan H., Wang H.-G., Lee S.-Y., and Pitt G.S.. 2014. Structural analyses of Ca^2+^/CaM interaction with NaV channel C-termini reveal mechanisms of calcium-dependent regulation. Nat Commun. 5:4896. doi:10.1038/ncomms5896. 25232683 PMC4170523

[R68] Wankowicz S.A., Ravikumar A., Sharma S., Riley B.T., Raju A., Flowers J., Hogan D., van den Bedem H., Keedy D.A., and Fraser J.S.. 2024. Uncovering Protein Ensembles: Automated Multiconformer Model Building for X-ray Crystallography and Cryo-EM. bioRxiv. 2023.06.28.546963. doi:10.1101/2023.06.28.546963. PMC11192534 38904665

[R69] Wayment-Steele H.K., Ojoawo A., Otten R., Apitz J.M., Pitsawong W., Hömberger M., Ovchinnikov S., Colwell L., and Kern D.. 2023. Predicting multiple conformations via sequence clustering and AlphaFold2. Nature. 1–3. doi:10.1038/s41586-023-06832-9. PMC10808063 37956700

[R70] Wilson C.J., Choy W.-Y., and Karttunen M.. 2022. AlphaFold2: A Role for Disordered Protein/Region Prediction? International Journal of Molecular Sciences. 23:4591. doi:10.3390/ijms23094591. 35562983 PMC9104326

[R71] Wisedchaisri G., Tonggu L., Gamal El-Din T.M., McCord E., Zheng N., and Catterall W.A.. 2020. Structural Basis for High-Affinity Trapping of the NaV1.7 Channel in Its Resting State by Tarantula Toxin. Molecular Cell. doi:10.1016/j.molcel.2020.10.039. PMC8043720 33232657

[R72] Wu X., and Hong L.. 2021. Calmodulin Interactions with Voltage-Gated Sodium Channels. Int J Mol Sci. 22:9798. doi:10.3390/ijms22189798. 34575961 PMC8472079

[R73] Xu H., Li T., Rohou A., Arthur C.P., Tzakoniati F., Wong E., Estevez A., Kugel C., Franke Y., Chen J., Ciferri C., Hackos D.H., Koth C.M., and Payandeh J.. 2019. Structural Basis of Nav1.7 Inhibition by a Gating-Modifier Spider Toxin. Cell. 176:702–715.e14. doi:10.1016/j.cell.2018.12.018. 30661758

