# Supplementary material for: Exploring voltage-gated sodium channel conformations and protein-protein interactions using AlphaFold2
Source: bioRxiv. 2024 Oct 18:2024.10.15.618559. Preprint. [Version 1] doi: 10.1101/2024.10.15.618559 (PMC11507785; doi:10.1101/2024.10.15.618559)

bioRxiv preprint doi: https://doi.org/10.1101/2024.10.15.618559; this version posted October 18, 2024. The copyright holder for this preprint (which was not certified by peer review) is the author/funder, who has granted bioRxiv a license to display the preprint in perpetuity. It is made available under aCC-BY 4.0 International license.

Supplemental Material Legends

Figure S1. Modeling of hNa$_V$1.1 with β-auxiliary subunits. (A-D) Superimposition of top ranked AlphaFold models of hNa$_V$1.1 in complex with the four β-auxiliary subunits with the corresponding experimental structure of reference: (A,B) hNa$_V$1.7-β1-β2 complex, PDB: 7W9K (Huang et al., 2022a); (C) hNaX- β3 complex, PDB: 7TJ8 (Noland et al., 2022); (D) hNa$_V$1.1-β4 complex, PDB: 7DTD (Pan et al., 2021). (E-H) Comparison of the distribution of states of the VSDs (E), IFM motif (F), AG (G), and SF (H) in the presence of the four different β-subunits and when the α-subunit is modeled alone.

Figure S2. Effect of the presence of CaM in α-subunit conformational distribution. (A) Comparison of the distribution of states of the VSDs for CaM bound to hNa$_V$1.2 (i) and hNa$_V$1.5 (ii) and the α-subunits modeled alone. (B) Comparison of the distribution of states of the SF and AG for CaM bound to hNa$_V$1.2 (i, ii) and hNa$_V$1.5 (iii, iv) and the α-subunits modeled alone.

Table S1. Definition of atom pairs to calculate the distances to use as state coordinates for each region of interest for each of the nine hNa$_V$ channels and hNaX.

Table S2. Reference values of distance coordinates for each region in experimental structures of human Na$_V$ **channels.** Other non-human structures are included to use as reference for particular states. All distances are in Å.

Video S1. Morphing between the most deactivated and most activated models for each of the VSDs.

Video S2. Morphing between the most contracted and most dilated SF conformations obtained in our models.

Video S3. Morphing between different states of the AG: (i) a closed state, (ii) the most open state obtained, (iii) an inactivated state, and (iv) returning to the closed state.

Video S4. Morphing across the six recycles of the top models of hNa$_V$1.7 bound to the four β-auxiliary subunits.

Video S5. Morphing between the most deactivated VSDIV state, obtained in the presence of β-3, and the most activated state.

Video S6. Morphing between the three identified states of the SF, being the same as Video S2 plus the new contracted state found in the presence of β-4.

Video S7. Morphing across the 6 recycles of the top model of CaM bound to hNa$_V$1.2. Only CaM and the C-T of the α-subunit are shown for clarity.

bioRxiv preprint doi: https://doi.org/10.1101/2024.10.15.618559; this version posted October 18, 2024. The copyright holder for this preprint (which was not certified by peer review) is the author/funder, who has granted bioRxiv a license to display the preprint in perpetuity. It is made available under aCC-BY 4.0 International license.

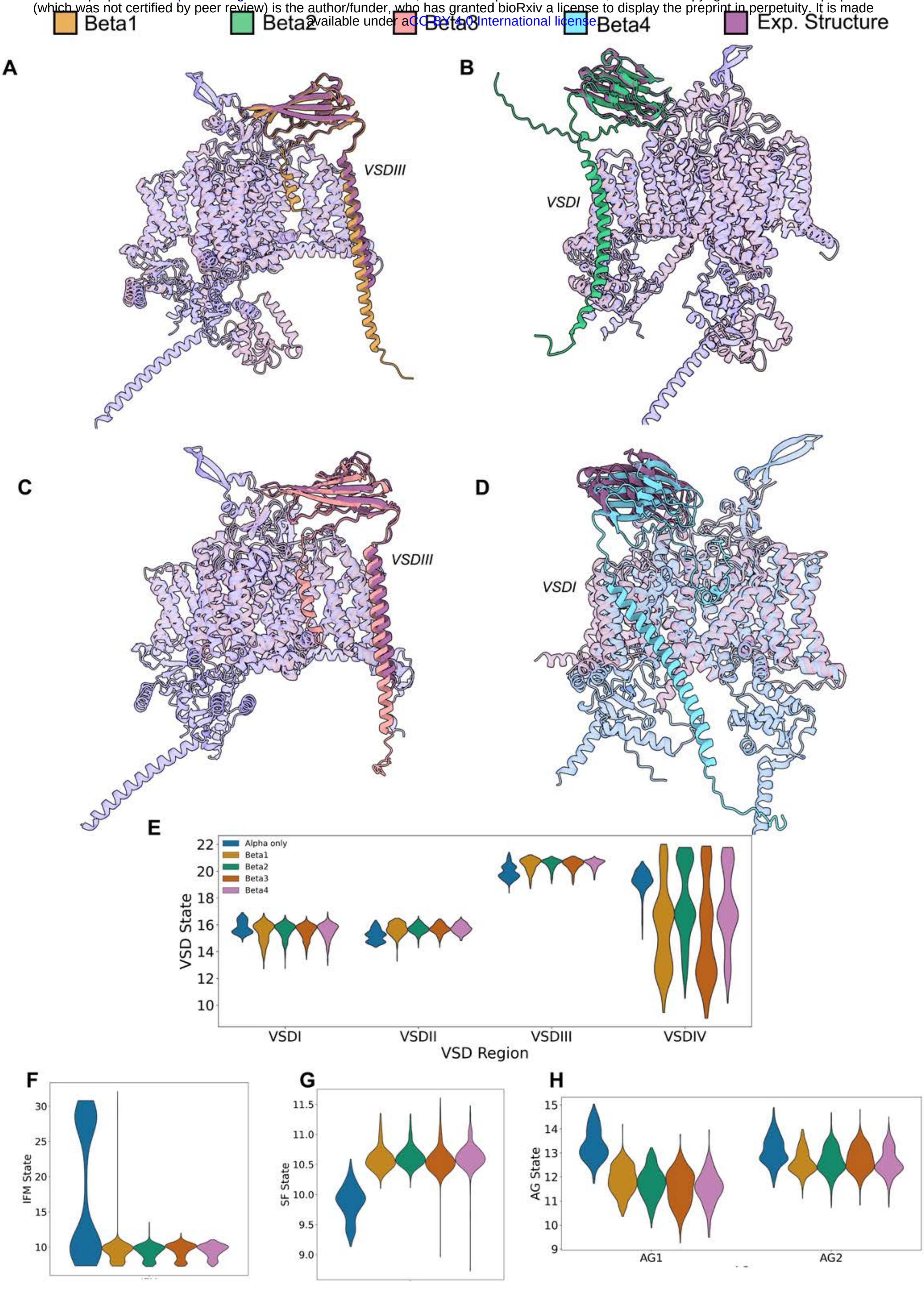

bioRxiv preprint doi: https://doi.org/10.1101/2024.10.15.618559; this version posted October 18, 2024. The copyright holder for this preprint (which was not certified by peer review) is the author/funder, who has granted bioRxiv a license to display the preprint in perpetuity. It is made available under aCC-BY 4.0 International license.

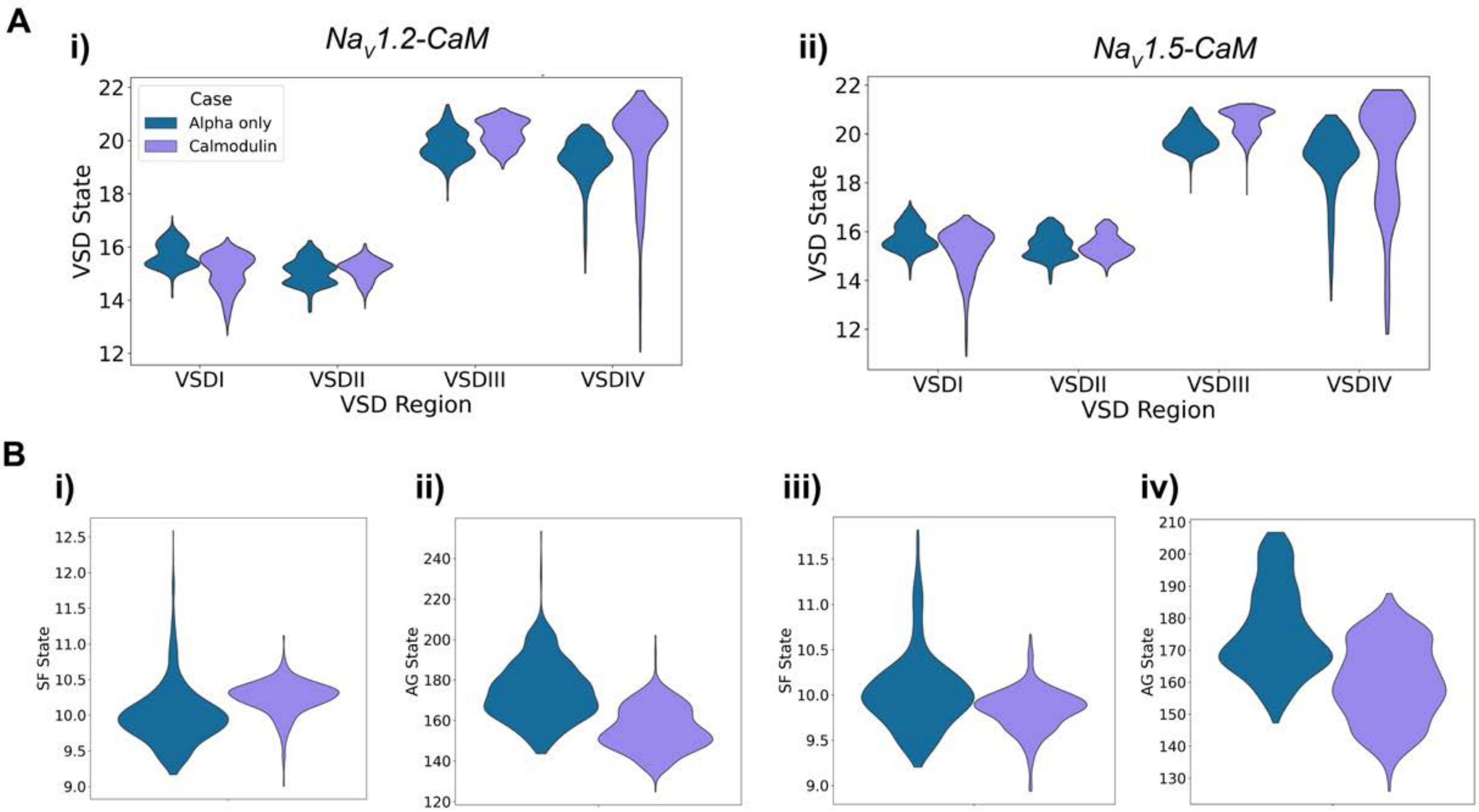

Supplement: Supplement 1 [file NIHPP2024.10.15.618559v1-supplement-1.pdf]
